# A History of Channel Coding in Aeronautical Mobile Telemetry and Deep-Space Telemetry

**DOI:** 10.3390/e26080694

**Published:** 2024-08-16

**Authors:** Michael Rice

**Affiliations:** Department of Electrical and Computer Engineering, Brigham Young University, Provo, UT 84602, USA; mdr@byu.edu

**Keywords:** block codes, convolutional codes, turbo codes, LDPC codes, telemetry

## Abstract

This paper presents a history of the development of channel codes in deep-space telemetry and aeronautical mobile telemetry. The history emphasizes “firsts” and other remarkable achievements. Because coding was used first in deep-space telemetry, the history begins with the codes used for *Mariner* and *Pioneer*. History continues with the international standard for concatenated coding developed for the *Voyager* program and the remarkable role channel coding played in rescuing the nearly-doomed *Galileo* mission. The history culminates with the adoption of turbo codes and LDPC codes and the programs that relied on them. The history of coding in aeronautical mobile telemetry is characterized by a number of “near misses” as channel codes were explored, sometimes tested, and rarely adopted. Aeronautical mobile telemetry is characterized by bandwidth constraints that make use of low-rate codes and their accompanying bandwidth expansion, an unattractive option. The emergence of a family of high-rate LDPC codes coupled with a bandwidth-efficient modulation has nudged the aeronautical mobile telemetry community to adopt the codes in their standards.

## 1. Introduction

Telemetry refers to the process of obtaining physical measurements and transmitting those measurements to another place for recording, display, and analysis. In aeronautical mobile telemetry, the measurements are obtained on an airborne test article and transmitted to the ground using an RF link. The data are used by flight test engineers to assess the performance of the airborne test article. In deep-space telemetry, the measurements are obtained on a space probe and include internal engineering measurements and scientific measurements. The measurements are transmitted to Earth using an RF link over enormous distances. The engineering data are used by engineers to monitor to health of the space probe; the science data are used by scientists to explore a variety of planetary and cosmic phenomena.

Aeronautical mobile telemetry emerged first. Deep-space telemetry had to wait until technological advances made it possible to send a probe into interplanetary space. The two telemetry systems were initially very similar. Over time, differences have emerged as the constraints placed on the two telemetries pushed them in different directions. Despite the fact that deep-space telemetry arrived on the scene after aeronautical mobile telemetry, deep-space telemetry adopted channel coding decades before aeronautical mobile telemetry.

This paper tells the story of channel codes in both telemetry applications. To limit the page count, the scope of this article is restricted to the use of channel codes in the RF links. Important contributions of coding to recording using magnetic tape and solid-state memory are not described in this article. In addition, the use of channel coding in Earth satellites is also not described. These histories will have to wait for another article to be written.

This article is organized as follows. After reviewing the early history and definitions in Section 2, the history of channel coding in deep-space telemetry is told in Section 3. The history of channel coding in aeronautical mobile telemetry follows in Section 4.

In the description of the channel codes, the following notation is used: An (n,k) block code maps *k* information bits to *n* coded bits where k<n. The block length of the code is *n*, the dimension of the block code is *k*, and the code rate is k/n. A rate-*r* constraint length *K* convolutional code is denoted by the (K,r) convolutional code.

## 2. Early History and Definitions

What is today known as aeronautical mobile telemetry originated during World War II (1939–1945) with the introduction of new fixed-wing aircraft and, at the end of the war, ballistic missiles [1,2]. Before deploying new aircraft or missiles, they had to be tested. Testing an experimental aircraft or missile involved instrumenting it with sensors to obtain in-flight measurements of key parameters. The measurements were radioed to the ground during the test flight. On the ground, flight test engineers monitored the received measurements. The main purpose of the test flight was to produce the measurements. Instrumented aircraft and missiles became known as *test articles*. Over time, test articles were expanded to include helicopters, guided missiles, and even artillery shells.

The measurements (the *telemetry*) transmitted to the ground (*telemetered*) included parameters such as structural stresses and strains, force, pressure, and temperature [2]. The sensors for each of these produced continuous-time outputs. One of the first issues in aeronautical mobile telemetry was the multiplexing problem. Instead of applying each sensor output to its own RF carrier, it quickly became obvious that tremendous savings in size, weight, and power could be achieved by using the sensor outputs to modulate *subcarriers*, in the 1–100 kHz range. The sum of the modulated subcarriers modulated the RF carrier. Because there were multiple options for doing this, the following notation was adopted to describe the option [3]:

*[multiplexing method]/[RF modulation]*

By the early 1950s, FM/FM was firmly established as the most common method for multiplexing continuous-time measurements and transmitting the multiplexed signal to the ground.

Over time, test articles became more sophisticated. More sophisticated test articles required more measurements to test them. Throughout the 1950s, it became increasingly common for the number of measurements to overwhelm the capacity of the FM/FM system. To address this problem, discrete-time multiplexing methods started to be used [3]. By 1960, analog-to-digital converters that quantized both the time axis (sampling) and the amplitude axis using an *N*-bit binary code (quantization) were viable for multiplexing sampled and quantized sensor outputs. When each of the *N* bits in the binary code is represented by a pulse, the resulting representation of the sample is called pulse code modulation (PCM). In fact, the first edition of the IRIG 106 standards dedicated exclusively to telemetry (published in 1960) contained a PCM standard [4] (see also [5]).

**Figure 1:**
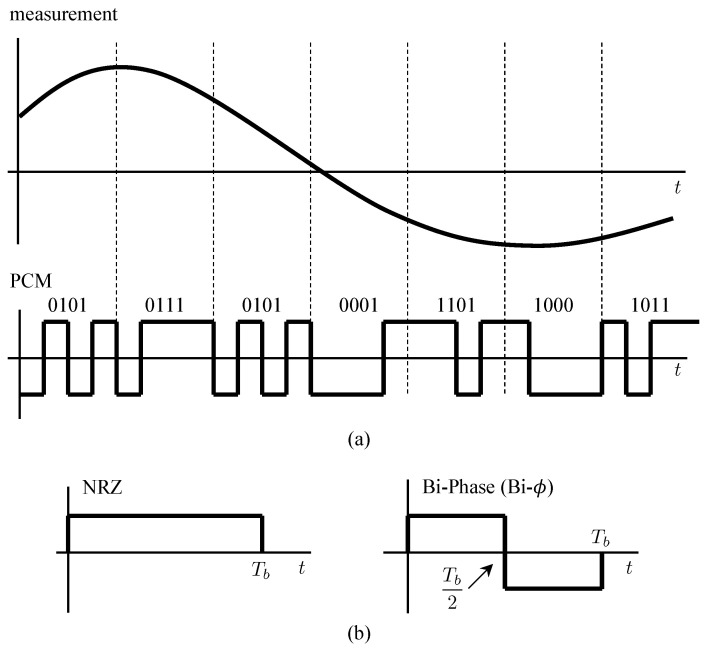
An illustration of pulse code modulation (PCM): (**a**) the continuous-time sensor output and the corresponding 4-bit PCM representation using the NRZ pulse; (**b**) the two most common pulses used to represent bits: the non-return-to-zero (NRZ) pulse and the bi-phase (bi-ϕ) pulse.

An example of a PCM system is illustrated in Figure 1a. A PCM system samples each continuous-time sensor output and represents the sample as an *N*-bit word. The words from different sensors are time-multiplexed on a word-by-word basis. Each bit in the *N*-bit word is represented in the waveform domain by a pulse. The two most common pulses used in telemetry are the non-return-to-zero (NRZ) pulse and the bi-phase (bi-ϕ) pulse. These pulses are illustrated in Figure 1b. In space telemetry, the NRZ pulse tended to be used when the telemetry data modulated a low-frequency subcarrier; the bi-ϕ pulse tended to be used when the telemetry data directly modulated the RF carrier. Initially, the same was true in aeronautical mobile telemetry, but the additional bandwidth required to accommodate the bi-ϕ pulse rendered its use increasingly rare as spectral congestion placed a premium on bandwidth.

**Figure 2:**
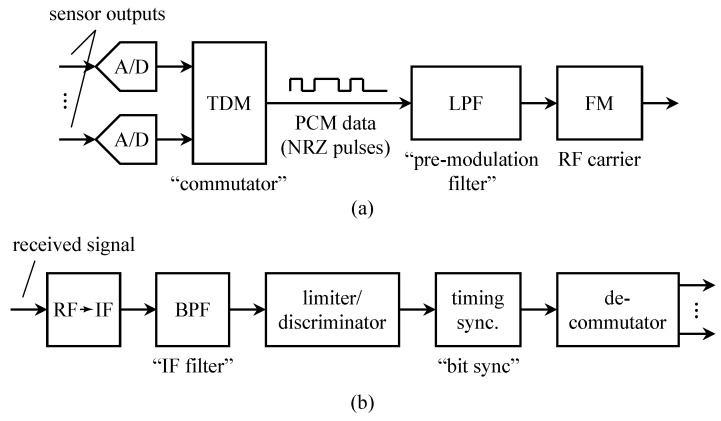
A block diagram illustrating PCM/FM used in aeronautical mobile telemetry: (**a**) the airborne multiplexer/transmitter; (**b**) the ground-based demodulator/demultiplexer.

In aeronautical mobile telemetry, the time-multiplexed PCM signal replaced the subcarrier FM signals to produce PCM/FM. A block diagram of a typical PCM/FM system is shown in Figure 2a. The multiplexing step is notional, if the A/D converter was fast enough, a single A/D converter was used with a commutator that switched the various continuous-time signals to the A/D converter input in a round-robin fashion. Over time, some of the inputs became “digital” in nature and did not require an A/D converter. Even so, the “digital” words could be multiplexed with the A/D outputs. To help address spectral congestion on busy test ranges, the bandwidth of the PCM/FM signal was reduced by inserting a low pass filter between the PCM data stream and the FM modulator. The low pass filter was called a pre-modulation filter.

A block diagram of the corresponding ground station is shown in Figure 2b. Here, the received signal is demodulated using a limiter/discriminator. The output of the limiter/discriminator is a noisy version of the low-pass filtered PCM signal. The limiter/discriminator output was viewed as a binary baseband PAM signal and applied to a binary baseband PAM detector comprising a detection filter (usually an integrate-and-dump operation) and a timing PLL to recover the PCM bits [3]. The words representing the quantized values of the sensor outputs were recovered using a de-commutator.

**Figure 3:**
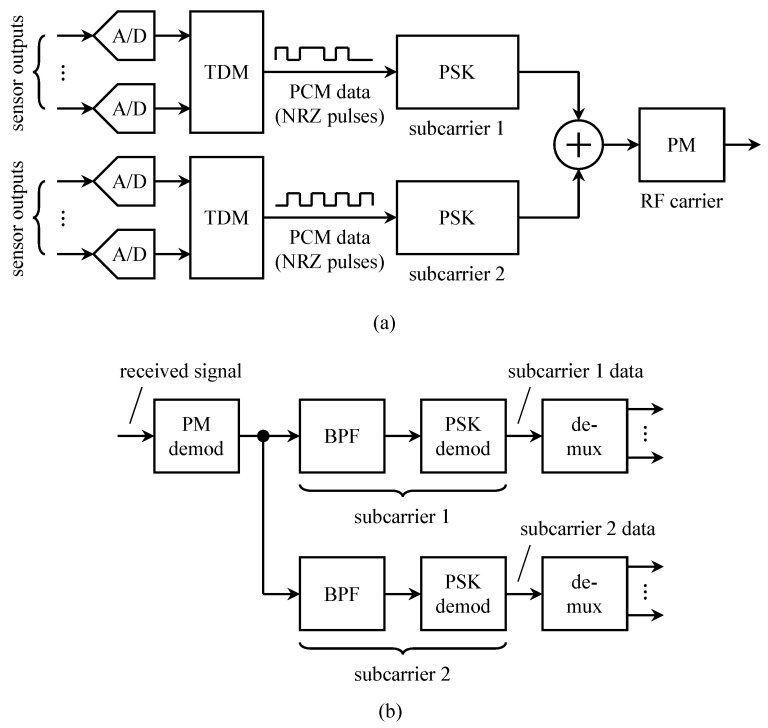
A block diagram illustrating PCM/PSK/PM used in aerospace telemetry: (**a**) the space based multiplexer/transmitter; (**b**) the ground-based demodulator/demultiplexer.

In deep-space telemetry, PCM/PSK/PM was quickly adopted as the preferred multiplexing/transmission method in the 1960s. This is because the performance advantage of coherent detection over non-coherent detection was required to close the space-to-Earth link. An illustration of a typical configuration for space telemetry is shown in Figure 3a. The earliest space probes using PCM typically had two or more telemetry streams in operation. A common configuration dedicated one data stream to “engineering telemetry data” and another to “scientific telemetry data”. The data rate for the engineering telemetry data was usually much lower than that of the scientific telemetry data. The data stream was itself composed of time-multiplexed data words. The telemetry data streams were frequency multiplexed by applying each one to a different subcarrier as shown. The subcarrier modulation was PSK. The PCM/PSK subcarriers were combined and used to phase-modulate (PM) an RF carrier. The PM modulation index was usually less than π/2 radians to produce a residual carrier component at the PM demodulator output [6]. The residual carrier component was used by the phase synchronizer to achieve lock when the signal-to-noise ratio was very low [7].

A block diagram of the corresponding ground station is shown in Figure 3b. The first step was to apply a coherent PM demodulator to the received signal. A coherent phase reference was extracted from the received signal as explained in the previous paragraph. Coherent PSK demodulators—comprising a matched filter (usually an integrate-and-dump operation), a carrier phase PLL, and a timing PLL—were applied to each subcarrier signal. The output bits were then de-multiplexed to access the data words for each sensor.

As a truly “digital” multiplexing technique, PCM was extremely flexible; it accommodated a wide variety of multiplexing requirements with relative ease. In addition, PCM offered the “digital advantage” of noise immunity. For example, in 1965 Stampfl, in reference to the Nimbus I weather satellite, observed [8]

*[O]nly an ultimate data accuracy of 4–5% on the 5-channel IR data could be achieved primarily due to tape recorder properties. Conversion to binary form prior to recording offers the only convenient means for accuracy improvement.*

Each additional bit added to the binary code representing the sample amplitude increases the signal-to-noise ratio of the sample by 6 dB [9]. For aeronautical mobile telemetry, it is relatively easy for data samples using a modest number of bits to achieve a higher signal-to-noise ratio than that achieved by FM/FM transmission followed by (RF) FM demodulation, (subcarrier) FM demodulation, and magnetic tape recording of the subcarrier FM demodulator output [10].

The “digital” nature of PCM allowed channel coding to be applied in a straightforward way. Because space telemetry adopted PCM in the early 1960s, telemetry with channel coding began in 1967. In contrast, it took over a decade for PCM/FM to replace FM/FM as the dominant multiplexing/transmission method in aeronautical mobile telemetry. This was due in part to the long life of weapons systems—systems designed in the FM/FM days were periodically tested over the next 20 or more years thus compelling ranges to maintain the ability to process FM/FM telemetry transmissions. Full-on adoption of PCM/FM also had to wait for advances in LSI to make PCM multiplexing smaller, faster, and cheaper than subcarrier FM multiplexing [8,10]. This issue was particularly important to missile systems, where size, weight, and power are limited. In the end, the advantages of PCM over existing techniques used in the 1950s and 1960s compelled telemetry system designers to adapt it for new test programs. By 1974, PCM/FM was the dominant multiplexing/modulation method used in aeronautical mobile telemetry [2]. A fascinating personal account of the transition is documented in [11]. Aeronautical mobile telemetry in Germany [12] and France [13] followed similar, but slightly different paths.

While both space and aeronautical mobile telemetry were poised to adopt channel coding at about the same time, channel coding was adopted much earlier in space telemetry. The reasons for the delayed adoption of aeronautical mobile telemetry are complex and nuanced, but at a high level may be attributed to trust, differences between the deep-space channel and the aeronautical mobile telemetry channel, and the role of encryption in aeronautical mobile telemetry.

**Trust**. Trust refers to the reluctance on the part of the customer to trust channel coding. This lack of trust derives from multiple sources. First, is the way decoders make errors in challenging environments. Many decoders for block codes produce decoding “failures” rather than an incorrect codeword. This feature sometimes leads customers to conclude that the code is “eliminating” data. The same thing happens with check bits, usually produced by cyclic redundancy check (CRC) codes: the hardware that performs the check usually deletes the data bits corresponding to the failed check bits. In contrast, a convolutional decoder using maximum likelihood sequence detection (usually performed by the Viterbi algorithm) always produces a sequence at the output. Sometimes, the output sequence is the wrong sequence. Customers often view an incorrect sequence as “worse” than the noisy version of an uncoded transmission. Second, customers are remarkably risk-averse. The conservative nature of the aeronautical mobile telemetry community produces an environment where customers are reluctant to approve the use of “new” technology in the testing phase of program development. This is one of the reasons why uncoded PCM/FM has remained so common.

Trust was an issue in the early days of space telemetry. The lack of trust was the reason the quick look-in property was imposed on the codes used in the *Pioneer* program (see Section 3.1). Trust remained an issue in aeronautical mobile telemetry for much longer [14]. For example, the customer did not approve the use of a simple CRC-based error-checking system, called the “frame thrower”, in the late 1990s (see Section 4.1).

**Deep-space and aeronautical mobile telemetry channels**. The deep-space channel and the aeronautical mobile telemetry channel have much in common. But the differences between the two have a bearing on the adoption of coding. The deep-space channel is characterized by its power constraint—extremely low power at the receiver input. This is caused by the enormous distances between the transmitter and receiver, often measured in multiples of the astronomical unit (1 AU = $149.6\times 10^{6}$ km). Consequently, deep-space links operate at low bit rates and must rely on low-rate channel codes to close the link. The corresponding increase in bandwidth due to the use of a low-rate channel code is an accepted cost.

In contrast, the aeronautical mobile telemetry channel is characterized by its bandwidth constraint. Because aeronautical mobile telemetry originates near the Earth’s surface, interference and channelization constraints are placed on aeronautical mobile telemetry signals in a way they are not for space telemetry. The increasing complexity of test articles increased the bit rate requirement for the telemetry downlink. This is the same trend that overwhelmed the capacity of FM/FM systems in the 1950s mentioned above. By 1969, the primary constraint in telemetry system design was bandwidth [15]. The bandwidth problem was exacerbated by a series of auctions that reassigned spectrum *from* aeronautical mobile telemetry *to* commercial services [16]. For a fixed information bit rate, channel coding increases bandwidth. Thus, for a channel code to be viable in aeronautical mobile telemetry, the performance improvement due to the channel code must be seen to be worth the cost of compounding the bandwidth problem.

Consequently, a good channel code for aeronautical mobile telemetry is a high-rate code coupled with a bandwidth-efficient modulation. High-rate channel codes are not as powerful as the low-rate channel codes that prevail in deep-space telemetry. The corresponding increase in required bit energy at the receiver is the accepted cost.

An additional characteristic of aeronautical mobile telemetry is link loss. The loss comprises a small static component defined by losses of waveguides, cables, connectors, radomes, etc., and a time-varying component due to a variety of factors such as disadvantageous propagation from the test article and multipath interference for low-elevation angle propagation. The time variations of the loss cause link outages that produce error bursts that can be quite long. The hope has been that channel coding would correct the long error bursts. Unfortunately, the extraordinarily long interleavers required to break up the long error bursts introduce an unacceptably long transmission delay.

**Figure 4:**
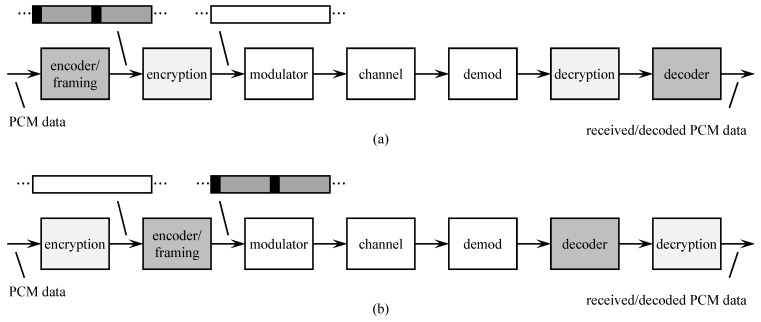
An encrypted aeronautical mobile telemetry system: (**a**) the encryption follows encoding; (**b**) encryption precedes encoding.

**Encryption**. When encryption was introduced to aeronautical mobile telemetry, the requirement was for encryption to be the “last thing” applied to the data before modulation. The arrangement is shown in Figure 4a. The reason this arrangement was required is that PCM framing and channel encoding both insert markers, in the form of known synchronization patterns, and in the data. The codeword markers are indicated by the black fields in the figure. Encryption worked best when there was no structure (i.e., markers) present in the encrypted data stream. The impact of this arrangement on channel coding was profound. First, because decryption had to be performed before decoding could be performed, the demodulator had to make hard decisions (the decryptor requires binary inputs). This removed the possibility for soft decision decoding. Second, errors in the demodulator output (the decryptor input) tend to cause long bursts of errors in the decryptor output. It is usually the case that the error bursts exceed the error-correcting capability of the hard decision decoding algorithm.

Later, the no-structure requirement was relaxed to allow markers to be inserted in the encrypted data stream. This opened the door for the arrangement shown in Figure 4b to be used. Here, encryption precedes encoding, thus placing the encoder as the last thing before modulation. This allowed the decoder to be the first thing after demodulation which is required for soft-decision decoding. The re-ordering also reduced the bit error probability at the decryptor input thereby reducing the probability of decryption introducing long error bursts into the PCM data.

In summary, while deep-space applications were in a position to use channel codes almost immediately after the appearance of PCM, the applications in aeronautical mobile telemetry occurred later. For this reason, the history of deep-space telemetry is described first, followed by the history of aeronautical mobile telemetry.

## 3. Deep-Space Telemetry

The *Pioneer* and *Mariner* space programs were nearly simultaneous exploration programs with complementary program goals. Each achieved a number of “firsts” in space exploration. Because the earliest (attempted) launches carried *Pioneer* spacecraft, the history of channel coding begins with the *Pioneer* program. History emphasizes channel coding “firsts” and remarkable missions where channel coding played an enabling role.

### 3.1. Pioneer

The *Pioneer* program comprised a series of unmanned spacecraft that monitored interplanetary phenomenon well outside the influence of the Earth. The missions designated *Pioneer* 6, 7, 8, and 9, launched 1965–1968, monitored interplanetary space weather in the inner solar system. *Pioneer* 10, launched in 1972, performed a Jupiter fly-by before heading into interstellar space. *Pioneer* 11, launched in 1973, performed a Saturn fly-by before heading into interstellar space.

The earliest *Pioneer* missions did not use any form of channel coding. The *Pioneer* 6 and 7 telemetry links used 6-bit data words plus a single parity bit to identify errors in the data words [17]. Erroneously received data words were discarded. Based on the studies described in [18], a systematic (25,12) convolutional encoder was installed on *Pioneer* 9 on an experimental basis [19]. The *Pioneer* 9 convolutional code was the first code to “fly in space” [20]. The convolutional code experiment was so successful that convolutional coding was planned for future *Pioneer* missions [19]. A more powerful (32,12) convolutional code was used for *Pioneer* 10 and 11 [20,21].

**Figure 5:**
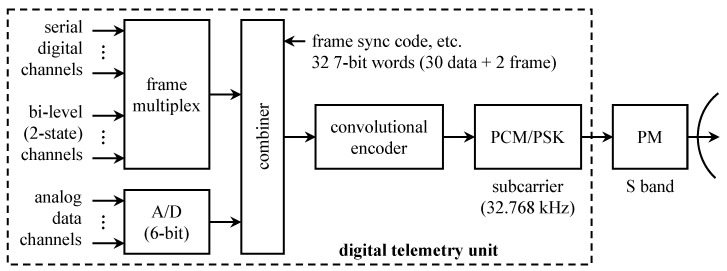
A block diagram of the digital telemetry unit and transmitter used for *Pioneer* 9, 10, and 11. Adapted from [21].

A simplified block diagram of the digital telemetry unit and transmitter for *Pioneer* 9, 10, and 11 is shown in Figure 5. There were three broad source categories: digital data, “bi-level” (two-state) data, and analog data. The analog data were sampled using a 6-bit A/D converter. The bits representing the quantized samples were combined with the bits from the other sources. Four different data formats and eight different bit rates (16 through 2048 bits/s in powers of 2) were supported depending on the phase of the mission [21]. The combined data formed the input to the convolutional encoder (optional for *Pioneer* 9). The convolutional code output modulated a subcarrier to produce a PCM/PSK signal. The PCM/PSK signal phase-modulated an S-band carrier at either 2292.03037 MHz or 2292.407407 MHz.

The convolutional codes used for *Pioneer* 9, 10, and 11 had long constraint lengths to achieve the desired coding gains. Given the technology constraints of the late 1960s the only practical decoder for these codes was the sequential decoding algorithm [22]. Consequently, the convolutional codes used in the *Pioneer* program were designed for sequential decoding. An additional constraint imposed on code design was the “quick-look-in” property [23]: the information bits appear explicitly in the encoded output. At the decoder input, the quick-look-in property makes it possible to obtain an estimate of the information bits prior to decoding. The justification for this property seems to be a mistrust of coding: a fear that coding might lose data that would otherwise not be lost [23].

**Figure 6:**
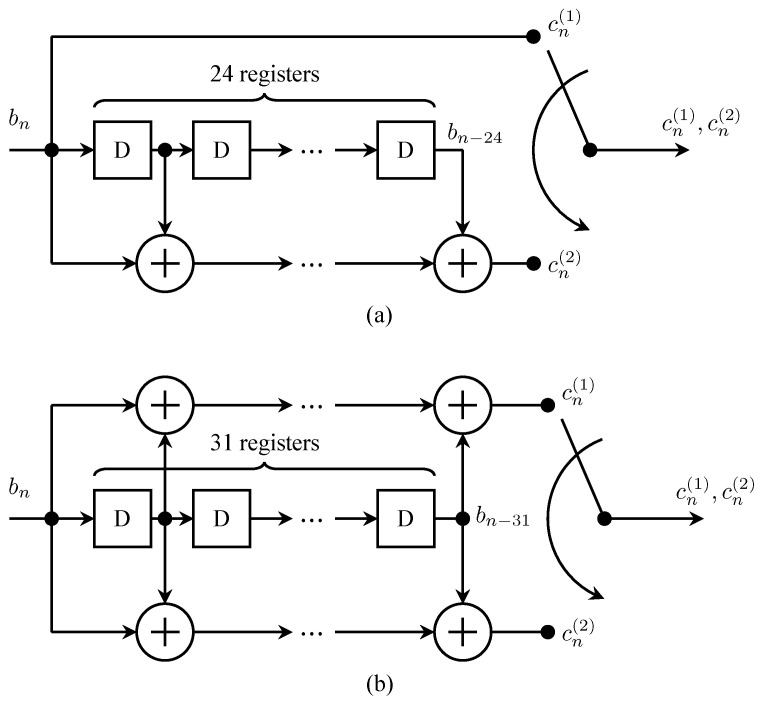
The rate-1/2 convolutional encoders used by the *Pioneer* program are as follows: (**a**) The systematic rate-1/2 convolutional encoder for *Pioneer* 9; (**b**) The non-systematic rate-1/2 convolutional encoder for *Pioneer* 10 and 11. The box with the “D” is a one-bit delay register.

Systematic convolutional codes possess the quick-look-in property by definition. For this reason, the first sequential-decoding-friendly convolutional codes considered for *Pioneer* 9 were systematic. The encoder for the *Pioneer* 9 convolutional encoders is shown in Figure 6a. The design criteria for this code are described in Section 3.3. For each input $b_{n}$, the encoder produced two outputs defined by the polynomials

(1) $$\begin{array}{cc} G^{\left(1\right)}\left(D\right) & =1\\ G^{\left(2\right)}\left(D\right) & =1+D+D^{3}+D^{5}+D^{7}+D^{8}+D^{11}+D^{13}\\ \; & \;+D^{14}+D^{15}+D^{19}+D^{20}+D^{21}+D^{23}+D^{24} \end{array}$$

The two coded bits are multiplexed to produce an output clocked at twice the rate as the input.

It is well-known that for a fixed constraint length, non-systematic convolutional codes are more powerful than systematic convolutional codes [20]. Consequently, the search for more powerful codes for *Pioneer* 10 and 11 focused on non-systematic does that possessed the quick-look-in property and that was well suited to sequential decoding. The (32,12) convolutional code from the class of codes developed in [23] was selected for *Pioneer* 10 and 11. The encoder for this code is shown in Figure 6b. For each input $b_{n}$, the encoder produced two outputs defined by the polynomials

(2) $$\begin{array}{cc} G^{\left(1\right)}\left(D\right) & =1+D+D^{2}+D^{4}+D^{5}+D^{7}+D^{8}+D^{9}\\ \; & \;+D^{11}+D^{13}+D^{14}+D^{16}+D^{17}+D^{18}+D^{19}+D^{21}\\ \; & \;\;+D^{22}+D^{23}+D^{24}+D^{25}+D^{27}+D^{28}+D^{29}+D^{31}\\ G^{\left(2\right)}\left(D\right) & =1+D^{2}+D^{4}+D^{5}+D^{7}+D^{8}+D^{9}\\ \; & \;+D^{11}+D^{13}+D^{14}+D^{16}+D^{17}+D^{18}+D^{19}+D^{21}\\ \; & \;\;+D^{22}+D^{23}+D^{24}+D^{25}+D^{27}+D^{28}+D^{29}+D^{31} \end{array}$$

The two coded bits are multiplexed to produce an output clocked at twice the rate as the input. The generator polynomials differ in only one position: $G^{\left(2\right)}\left(D\right)$ is missing the $D^{1}$ term of $G^{\left(1\right)}\left(D\right)$. This one-bit difference endows the non-systematic code with the quick-look-in property [23]. This code achieved a coding gain of approximately 6.9 dB.

Decoding for the *Pioneer* 9, 10, and 11 missions was performed in the ground station. The output of the coherent phase demodulator was applied to the integrate-and-dump detector in the subcarrier PSK demodulator. The output of the integrate-and-dump circuit was quantized to 3 bits [24]. The convolutional decoder performed sequential decoding [22] on the 3-bit quantized values. The decoding algorithm was modified to exploit the presence of known values embedded in the telemetry stream [17]. This modification added an extra layer of error detection.

### 3.2. Mariner

The *Mariner* program was one of the first efforts to send unmanned spacecraft to other planets. *Mariner* launched ten spacecraft (eight of which were successful) between 1962 and 1973 [25]. *Mariner* 2 and 5 explored Venus, *Mariner* 3, 4, 6, 7, and 9 explored Mars, and *Mariner* 10 explored both Venus and Mercury [25]. The telemetry link on *Mariner*s 1–5 used uncoded PCM/PSK/PM [26]. *Mariner* 6, 7, and 9, launched 1969–1971 used channel coding [27,28].

**Figure 7:**
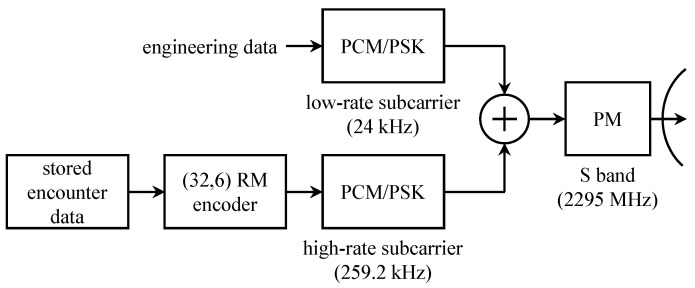
A block diagram of the *Mariner* 6 spacecraft.

A block diagram representing the telemetry subsystem on the *Mariner* 6 spacecraft is shown in Figure 7. The telemetry link comprised two subcarriers, each modulated using PCM/PSK. The subcarriers were combined, and the resulting combination phase-modulated an S-band carrier at 2295 MHz.

The low-rate subcarrier carried the engineering data: time-division multiplexed binary words representing samples of transducer outputs [26]. During the cruise phase, uncoded engineering data were transmitted on the subcarrier at either 3313 bits/s or 813 bits/s [27]. During image transmission, uncoded engineering data were transmitted to the subcarrier at either 6623 bits/s or 270 bits/s.

The high-rate subcarrier transmitted digitized images of Mars read back from tape storage. To capture the images, *Mariner* 6 was equipped with a TV camera whose (continuous-time) output was recorded to magnetic tape using an onboard tape recorder. After an imaging session, the analog tape was played back at a lower speed through an A/D converter. The A/D converter output was recorded on a second onboard tape recorder. The digital data comprised 6-bit samples representing the grayscale level (64 grayscale levels) for each pixel in the digitized image. The data stored on the digital tape recorder was played back at an even lower speed for coding and transmission [27]. The (32,6) bi-orthogonal Reed–Muller code was used to protect pixel values. The bit rate was 16.2 kbits/s [27].

The encoder is described by the 6×32 generator matrix [27].

(3) $$G=\left[\begin{array}{c} 1\;1\;1\;1\;1\;1\;1\;1\;1\;1\;1\;1\;1\;1\;1\;1\;1\;1\;1\;1\;1\;1\;1\;1\;1\;1\;1\;1\;1\;1\;1\;1\\ 0\;0\;0\;0\;0\;0\;0\;0\;0\;0\;0\;0\;0\;0\;0\;0\;1\;1\;1\;1\;1\;1\;1\;1\;1\;1\;1\;1\;1\;1\;1\;1\\ 0\;0\;0\;0\;0\;0\;0\;0\;1\;1\;1\;1\;1\;1\;1\;1\;0\;0\;0\;0\;0\;0\;0\;0\;1\;1\;1\;1\;1\;1\;1\;1\\ 0\;0\;0\;0\;1\;1\;1\;1\;0\;0\;0\;0\;1\;1\;1\;1\;0\;0\;0\;0\;1\;1\;1\;1\;0\;0\;0\;0\;1\;1\;1\;1\\ 0\;0\;1\;1\;0\;0\;1\;1\;0\;0\;1\;1\;0\;0\;1\;1\;0\;0\;1\;1\;0\;0\;1\;1\;0\;0\;1\;1\;0\;0\;1\;1\\ 0\;1\;0\;1\;0\;1\;0\;1\;0\;1\;0\;1\;0\;1\;0\;1\;0\;1\;0\;1\;0\;1\;0\;1\;0\;1\;0\;1\;0\;1\;0\;1 \end{array}\right].$$

The code word $c=[c_{0}\ldots ,c_{31}]$ corresponding to the data word $b=[b_{0},\ldots ,b_{5}]$ may be computed using vector/matrix multiplication c=bG where all arithmetic operations are performed in GF(2). The circuit used on *Mariner* to generate a codeword was different [27].

The Reed–Muller code was chosen because a computationally efficient decoding algorithm was available. The code comprises 64 length-32 codewords. The codewords can be viewed as a set of 32 orthogonal length-32 binary vectors together with their complements. For this reason, the (32,6) Reed–Muller code is sometimes called a “bi-orthogonal” code. Soft-decision decoding was performed using a correlator based on the Hadamard transform [29]. The coding gain for the *Mariner* 6 and 7 configurations was 2.2 dB [27].

**Figure 8:**
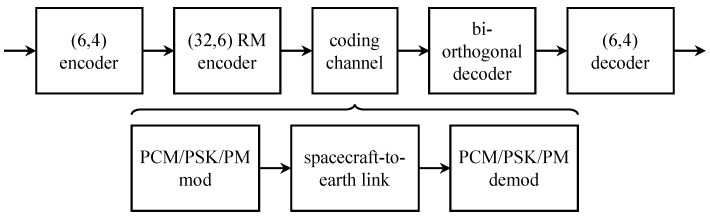
A block diagram of the concatenated coding system used for *Mariner* 9.

The last *Mariner* mission, *Mariner* 9, carried an infrared interferometer spectrometer (IRIS) experiment to provide information about the Martian atmosphere. Each measurement, called an interferogram, was sampled using 6-bit quantization to produce 4096 6-bit words. The middle 512 words of the interferogram contained essentially all of the interferogram information and required an equivalent bit error probability no more than $10^{-5}$. This was not achievable with the worst-case link conditions using the (32,6) Reed–Muller code alone. For this reason, a concatenated coding scheme illustrated in Figure 8 was used [30]. The outer code was a short (6,4) “generalized Hamming code” defined over GF($2^{6}$)—each 6-bit word was treated as a code element—with minimum distance $d_{\min}=3$ that was capable of correcting t=1 element error. The inner code was the (32,6) Reed–Muller code described above. The idea was that when the (32,6) Reed–Muller code made an error, the erroneous decoder output was the wrong 6-bit word. If only one Reed–Muller decoder error occurred in six consecutive words, then the outer code saw this as a single error and could correct it.

The field generator polynomial used for the (6,4) code was [30],

(4) $$F\left(X\right)=X^{6}+X+1$$

over GF(2). The generator matrix was as follows:

(5) $$G=\left[\begin{array}{cccccc} 1 & 1 & 1 & 0 & 0 & 0\\ 1 & \alpha & 0 & 1 & 0 & 0\\ 1 & \alpha^{2} & 0 & 0 & 1 & 0\\ 1 & \alpha^{3} & 0 & 0 & 0 & 1 \end{array}\right]$$

where α is a primitive element in GF($2^{6}$). Decoding was accomplished using syndrome decoding. The equivalent bit-level encoder and decoder are described in [30].

*Mariner* 9 was the first to use concatenated coding for space communications. Unlike later programs, such as *Voyager* 1 and *Voyager* 2, which used a convolutional code as the inner code, *Mariner* 9 used a block code as the inner code. What is common is the use of soft-decision decoding for the inner code.

### 3.3. The Story behind the Pioneer and Mariner Code Designs

Readers new to the field of deep-space communications should keep in mind that the design cycles for deep-space communications are very long. Planning and design start long before launch. Following best practices, the final design is “locked in” for thorough testing and debugging years before launch. This is why advancements in coding, published well before the launch date, were sometimes unavailable to the designers.

The first of the two codes designed was for *Mariner* 6/7. At the time, it was well-known that what is now called “soft decision decoding” was the best way to maximize coding gain [31]. Consequently, the first codes considered for *Mariner* 6/7 were convolutional codes.

At the time, the only known soft decision decoder was the sequential decoding algorithm developed by Wozencraft [32]. Sequential decoding models the convolutional code output as a tree and pursues branches in the tree that it believes are viable candidates for the decoded sequence. It pursues good branches as long as the branch is deemed good. If a previously pursued branch turns out to be a bad branch, the algorithm must back up and evaluate a different branch. The number of computations (and, hence, the amount of time) required to make a decision is variable and it depends strongly on channel conditions.

A prototype of Wozencraft’s sequential decoder, instantiated in custom hardware, had been tested over the dial-up telephone channel at MIT Lincoln Labs. The results were not promising and did not provide convincing evidence that convolutional codes with sequential decoding were a viable option for deep-space communications [31]. Consequently, the *Mariner* 6/7 team elected to use block codes. Fano’s sequential decoding algorithm [22], which accomplishes sequential decoding with fewer computations, had not yet been developed. Had Fano’s algorithm been available, it is possible the decision would have been different.

During the *Mariner* 6/7 design phase, there were few block codes with viable soft-decision decoding algorithms. Of the few codes available, the family of Reed–Muller codes was an attractive choice. The attraction was the simplicity of the encoder [27,33] and the use of the Hadamard transform to simplify soft-decision decoding [33]. For these reasons, the Reed–Muller code described in Section 3.2 was selected.

The *Pioneer* 9 team started the design effort in the mid-1960s, *after* the *Mariner* 6/7 team. By this time, Fano’s sequential decoding algorithm was known and it was believed that convolutional codes with sequential decoding were viable for space communications. Convolutional codes designed for sequential decoding have good column distance properties [34]. With such codes, different branches (i.e., code sequences) are (usually) quickly distinguishable at the beginning of the decoding process. An in-depth study by Lumb [18] identified a good candidate convolutional code for use on *Pioneer* 9. The search was an extension of the design procedure published by Lin and Lyne [35]. To bypass NASA’s lengthy qualification process, the recommended code was to be installed in *Pioneer* 9 as a “telemetry coding experiment” [24]. The search for codes was constrained as follows:

1. The code had to possess a good column distance profile for the reasons outlined in the previous paragraph.
2. The code rate was 1/2. Lower code rates reduced the received coded $E_{b}/N_{0}$ below the threshold where carrier phase and symbol timing synchronizers could operate.
3. The code had to be systematic because systematic codes possess the “quick-look-in” property described in Section 3.1. (Massey and Costello later showed that it is possible to construct non-systematic convolutional codes with the quick-look-in property [23]).
4. The constraint length was limited to 25. Due to the code’s status as an experiment, no hardware version of the decoder was developed. Instead, the decoder was programmed in software on the Scientific Data Systems (SDS) 920 mainframe computer [24]. The SDS 920 used 24-bit words [36]. To produce a decoder that operated in real-time (512 bits/s), the constraint length of the code was limited to 25 so that the copy of the 24-bit binary sequence defining the encoder memory fit into one 24-bit binary word on the SDS 920 [24].

The result was the code recommended in [17,18] and described by the polynomials (1) and illustrated in Figure 6a.

*Pioneer* 9 was launched in November 1968 **before** *Mariner* 6, thus, making its experimental convolutional code the first used in space. Following the standard operating procedure for experimental hardware, *Pioneer* 9 was launched with the encoder bypassed. In December 1968, the experimental encoder was activated [24] and remained active except for occasional passes where the only ground stations available were ones without a decoder. The experiment was so successful that convolutional encoders were planned for the next two missions, *Pioneer* 10 and *Pioneer* 11, on a non-experimental basis. As described in Section 3.1, a more powerful non-systematic (32,12) convolutional code described by the polynomials (2) and illustrated in Figure 6b was used for *Pioneer* 10 and 11. The code was also used in on the West German *Helios A* and *Helios B* solar orbiters [37,38]. In 1983, this code was believed to be the most widely used code in deep-space communications [33].

A postscript to this story is the 1967 publication of the Viterbi Algorithm (VA) [39]. Originally, the algorithm was thought to be “asymptotically optimal”. In 1973, Forney reformulated the VA as a trellis search (the form most familiar to contemporary readers) and showed that the VA performs maximum likelihood sequence detection (MLSD) [40]. In the context of deep-space communications, the advantages of the Viterbi algorithm were as follows: (1) MLSD came closer to unlocking the coding gain a convolutional code was capable of achieving, and (2) the number of computations (and therefore the decoding delay) is fixed. The number of computations required by the VA grows exponentially with constraint length. Consequently, the earliest codes to use MLSD decoding were called “short constraint length codes” (such as the constraint length-7 code used by *Voyager*) to distinguish them from the long constraint length codes intended for sequential decoding. Because VA decoding performs MLSD, good convolutional codes are those that have good free distance profiles. (This is in contrast to convolutional codes designed for sequential decoding where, as explained above, a good column distance is preferable). The prospect of MLSD decoding prompted the search for a different class of convolutional codes. A noteworthy early contribution is the 1970 dissertation by one of Viterbi’s UCLA PhD students, Odenwalder [41]. The (7,12) convolutional code used by *Voyager* [described by the polynomials (6) and illustrated in Figure 12 in the next section] was one of the codes published in Odenwalder’s dissertation. MLSD decoding essentially ended the use of sequential decoding for deep-space communications [33].

### 3.4. Viking

The Viking program sent two missions to Mars, both launched in 1975. Each mission comprised an orbiter and a lander. The lander separated from the orbiter and descended to a soft landing on the Martian surface. In its day, the Viking program was one of the most highly visible NASA programs [25]. It produced the first images of the surface of another plant *from the surface* of that planet and it was equipped with signs-of-life tests applied to soil samples scooped into test chambers in the lander.

**Figure 9:**
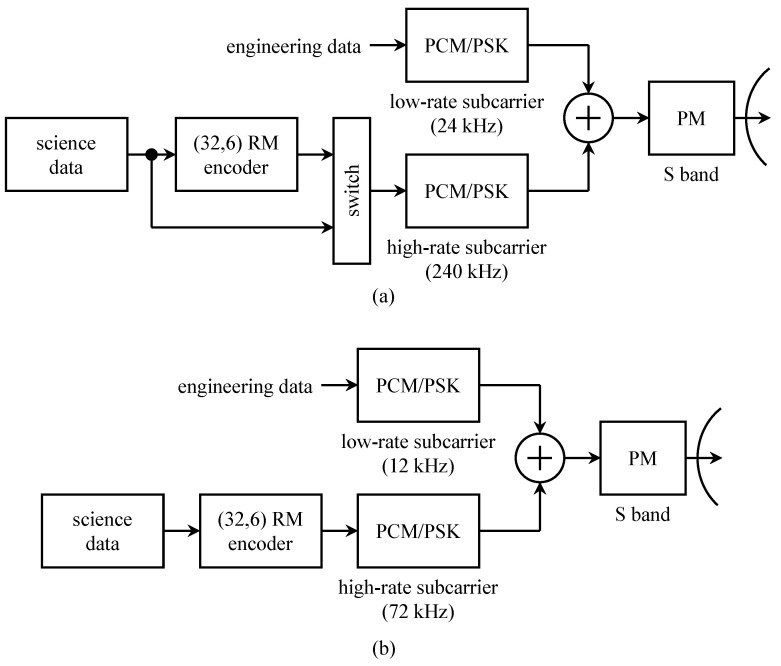
The Viking telemetry systems: (**a**) the orbiter; (**b**) the lander.

The telemetry system for the Viking orbiter is shown in Figure 9a. Uncoded engineering data, at a rate of 813 or 3313 bits/s, was used to modulate the low-rate subcarrier using PCM/PSK [42]. Science data were transmitted on a high-rate subcarrier using PCM/PSK and could be coded or uncoded. The uncoded data rate was 1, 2, or 4 kbits/s and the coded data rate was 1, 2, 4, 8, or 16 kbits/s [42]. The code was the same (32,6) Reed–Muller code used for the *Mariner* program described in Section 3.2. The two subcarriers were frequency division multiplexed and the combination was used to phase-modulate an S-band carrier at 2300 MHz [43,44].

The telemetry system for the Viking lander is shown in Figure 9b. The configuration shown here is for the direct Mars-to-Earth link. (Each lander was equipped with a 381 MHz UHF link to the orbiter [45,46]). The lander telemetry system is nearly identical to that of the orbiter, except the subcarrier frequencies and data rates were lower and there was no provision for uncoded transmission of science data. The engineering data rate was 813 bits/s and the coded science data rate was 250, 500, or 1000 bits/s [42].

### 3.5. Voyager

The *Voyager* program sent two unmanned spacecraft, each an advanced version of the *Mariner* spacecraft [47], to the outer solar system, and on to interstellar space. *Voyager* 1, launched in 1977, visited Jupiter and Saturn before leaving the solar system [48]. *Voyager* 2, also launched in 1977, visited Jupiter, Saturn, Uranus, and Neptune before leaving the solar system [48].

**Figure 10:**
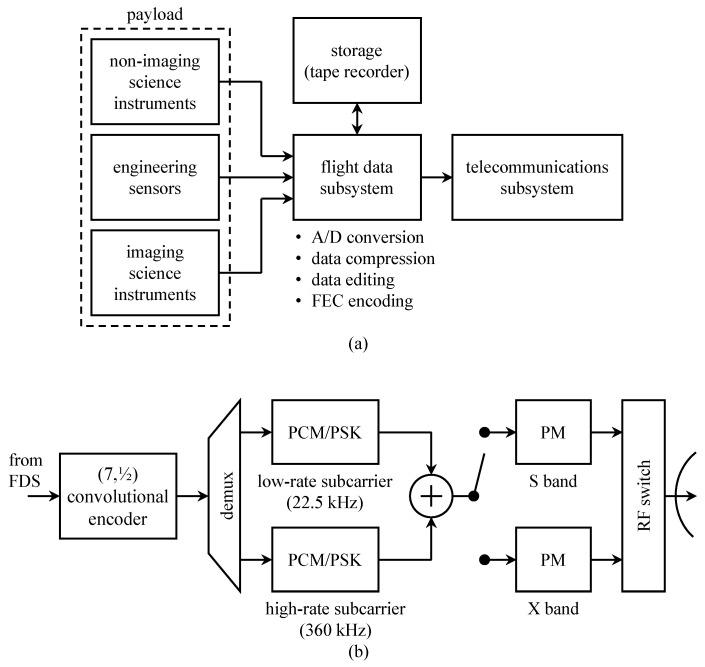
A block diagram of the *Voyager* 1 and 2 telemetry system: (**a**) a system-level overview (adapted from [47]); (**b**) a detailed view of the telecommunications subsystem.

A block diagram of the *Voyager* telemetry system is shown in Figure 10a. The payload comprises science instruments and engineering sensors. The flight data subsystem (FDS) sampled the continuous-time outputs of the instruments and sensors, compressed the image data (optional), encoded the data using one of the coding options described below, and arranged the data for subcarrier modulation. The data were either transmitted in real-time or stored for play-back transmission at a later time, possibly at a lower data rate.

Transmission was performed by the telecommunications subsystem. A detailed view of the telecommunications subsystem is shown in Figure 10b. For transmission, data from the FDS modulated one of two subcarriers [48]. The high-rate subcarrier was used for transmission rates greater than 7.2 kbits/s. The subcarriers were combined, and the resulting combination phase-modulated either an S-band carrier (at 2296.481481 MHz or 2295.5 MHz), or an X-band carrier (at 8420.432097 MHz or 8415 MHz) [48].

The telemetry bit rate was adjustable, depending on both the spacecraft-to-Earth distance, S-band, or X-band transmission, and the kind of data to be transmitted. The slowest mode transmitted uncoded engineering data at 10 b/s. The other modes were convolutionally encoded (described below) with information rates ranging from 10 b/s to 115.2 kbits/s.

**Figure 11:**
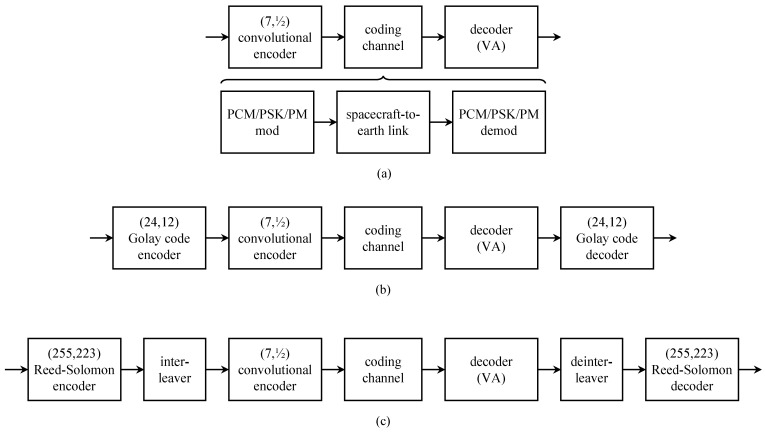
Coding procedures used for non-image telemetry data for *Voyager* 1 and 2: (**a**) (7,12) convolutional encoding; (**b**) concatenated coding using the (24,12) Golay code as the outer code and the (7,12) convolutional code as the inner code; (**c**) concatenated coding using the (255,223) Reed–Solomon code as the outer and the (7,12) convolutional code as the inner code.

The reliability of the non-imaging science instrument data and the engineering sensor data was a bit error probability no greater than $5\times 10^{-5}$ [47]. Given the relatively low data rates for these data, the desired reliability was achievable using the (7,12) convolutional code with soft-decision Viterbi decoding and with decreasing bit rates as the spacecraft traveled further from the Earth. This coding strategy is illustrated in Figure 11a. During planetary encounters, image data were also transmitted along with the engineering data. The required reliability for image data was a bit error probability of $5\times 10^{-3}$. Because 95% of the data were image data, it was more efficient to design the link, from convolutional encoder input to convolutional decoder output, to achieve a bit error probability of $5\times 10^{-3}$ and apply an additional layer of coding to the non-imaging data [47]. For the Jupiter and Saturn encounters, non-imaging science instrument data and the engineering sensor data were protected using the concatenated coding system shown in Figure 11b [49]. The outer code was the Golay (24,12) code and the inner code was the (7,12) convolutional code. The Uranus and Neptune encounters with *Voyager* 2 used the concatenated coding system shown in Figure 11c [49]. The outer code was the (255,223) Reed–Solomon and the inner code was the (7,12) convolutional code. The interleaver between the inner and outer code had a depth of four symbols.

**Figure 12:**
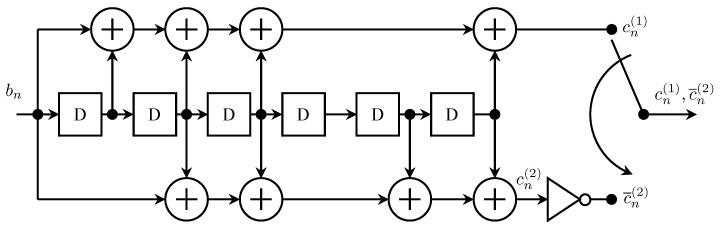
The encoder for the (7,12) convolutional code used by *Voyager* 1 and *Voyager* 2. Adapted from [6]. The box with a “D” is a single-bit delay.

The encoder for the (7,12) convolutional code is shown in Figure 12 [6]. This code is defined by the following polynomials:

(6) $$\begin{array}{cc} G^{\left(1\right)}\left(D\right) & =1+D+D^{2}+D^{3}+D^{6}\\ G^{\left(2\right)}\left(D\right) & =1+D^{2}+D^{3}+D^{5}+D^{6}. \end{array}$$

The code was originally discovered by Odenwalder in [41] and is characterized by a free distance $d_{\mathrm{free}}=10$. Maximum-likelihood sequence detection performed by the Viterbi algorithm (VA) was used for decoding. The decoder input was the soft-decision output of the subcarrier PCM/PSK demodulator using 3-bit quantization [6]. The VA operated on a trellis with $2^{6}=64$ states. The coded link achieved a bit error probability of $5\times 10^{-5}$ at $E_{b}/N_{0}=2.34$ dB. This code later became the NASA and CCSDS standard rate-1/2 convolutional code [6,50]. The standard code applies an inverter to $c_{2}^{\left(n\right)}$ to increase the bit transition density in the case of a long string of all ones or all zeros at the encoder input [51]. The inverter does not change the distance properties of the code. A high bit transition density improves the performance of decision-directed bit synchronizers.

The (24,12) Golay code was an extended version of the (23,12) Golay code. The (23,12) Golay code is a systematic cyclic code whose generator polynomial is [52]

(7) $$g\left(X\right)=X^{11}+X^{9}+X^{7}+X^{6}+X^{5}+X+1.$$

The (23,12) Golay code has a minimum distance $d_{\min}=7$ and can correct t=3 errors. To create the (14,12) Golay code, each 23-bit codeword was extended to 24 bits through the addition of a parity check bit. The extended Golay code has a minimum distance of 8 and, in addition to correcting 3-bit errors, can detect the presence of 4-bit errors. The decoder is based on the Berlekamp–Massey algorithm applied to the 23 bits corresponding to the codeword before extension [52].

The (255,223) Reed–Solomon code is a systematic code based on defined on GF($2^{8}$), i.e., each code symbol is an 8-bit symbol. The field generator polynomial is [50]

(8) $$F\left(X\right)=X^{8}+X^{7}+X^{2}+X+1$$

over GF(2). The code generator polynomial is [50]

(9) $$G\left(X\right)=\prod_{j=112}^{144}\left(X-\alpha^{11j}\right)$$

where $\alpha^{11}$ is a primitive element in GF($2^{8}$). The code has a minimum distance $d_{\min}=33$ symbols, which gives it an error-correcting capability of t=16 symbols.

The interleaver is a block interleaver with 4 rows and 255 columns. The codewords are input into the block interleaver row-by-row and output column-by-column [6]. At the ground station, the deinterleaver reverses the procedure: the output bits from the Viterbi decoder are organized into 8-bit symbols and read into the interleaver column-by-column. The deinterleaver contents are read out row-by-row and applied to the Reed–Solomon decoder. The purpose of the deinterleaver is to account for the fact that decoding errors in the inner decoder produce error bursts in the decoder output. The error bursts can be several constraint lengths long [6]. Without interleaving several long error bursts can occur in a single Reed–Solomon codeword [53]. In such a case, the error burst can overwhelm the error-correcting capability of the Reed–Solomon code [54]. The interleaver distributes the error bursts produced by the inner decoder over multiple Reed–Solomon codewords. The interleaver, inner encoder, channel, inner decoder, and deinterleaver cascade tends to present to the outer decoder a discrete memoryless channel [6]; just what the Reed–Solomon code was designed for.

This concatenated coding system was able to achieve a bit error probability of $1\times 10^{-6}$ at $E_{b}/N_{0}$ in the range of 2.5 to 3.0 dB. Because the achievable error rate was so low, lossless image compression was able to be applied to the image data encoded by the concatenated coded system [49].

### 3.6. Giotto

*Giotto*, the first deep-space mission conducted by the European Space Agency [55], was launched in July 1985 on a mission to explore Comet Haley. Because the exploration involved a high-velocity fly-by close the the comet nucleus, there was a high probability that the spacecraft would be destroyed. Consequently, the standard approach of measuring, storing, and then transmitting the measurements, was replaced by a system that transmitted the telemetry in real-time on an 8.428 GHz downlink [56]. The engineering telemetry link (called the “housekeeping” link by the *Giotto* team [57]) was 350 bits/s. The bit rate for each instrument is listed in Table 1 of [57]. The composite telemetry bit rates ranged from 360 bits/s to 46,080 bits/s in 4 selectable formats [56]. The channel code was the same concatenated coding scheme used by *Voyager* 2 illustrated in Figure 11c [56]: the outer code was the (255,223) Reed–Solomon code and the inner code was the rate (7,12) convolutional code.

The *Giotto* mission was a success; in March 1986 it flew within 610 km of the comet nucleus according to Wilkins [56] or at 596 km from the comet nucleus according to Doody [58] (p. 360) and survived the encounter. Because *Giotto* survived the encounter, it was directed to a fly-by of Comet Grigg-Skjellerup at 200 km in July 1992 [58].

### 3.7. Galileo

NASA’s *Galileo* mission comprised a sophisticated orbiter and atmospheric entry probe designed to explore Jupiter. The orbiter and probe were launched as a combined unit. The probe separated from the orbiter five months before arriving at Jupiter and was timed to plunge into the Jovian atmosphere just as the orbiter made its initial (and closest) pass by Jupiter. The probe transmitted its telemetry to the orbiter during the orbiter’s initial pass. After this, the orbiter maneuvered into orbit around Jupiter to explore Jupiter and its moons. This ambitious program suffered an extraordinary number of setbacks that tested the patience of the team [59]. Because these setbacks form the context for several developments in channel coding, a brief summary of the original plans, the setbacks, and the consequences of those setbacks is described first.

Initial planning in the 1970s called for a direct launch from Earth to leverage an advantageous Earth–Jupiter alignment. The direct launch was to be on a Centaur multi-stage rocket. The lower stages powered the upper stage into Earth’s orbit. The upper stage, equipped with its own rocket engine, carried the *Galileo* spacecraft as its payload. In 1975, NASA enacted a policy requiring all planetary missions to use the then-future Space Shuttle (the first launch was in 1981) for placing planetary exploration vehicles into Earth orbit. The Space Shuttle launch date for *Galileo* was scheduled for January 1982. The Space Shuttle cargo bay was to carry the Centaur upper stage containing the *Galileo* Spacecraft. The high-energy, liquid hydrogen-fueled Centaur upper stage was powerful enough to transition *Galileo* from Earth orbit to a direct trajectory to Jupiter; a voyage of 27 months. The launch date was delayed three times to May 1986 for causes not involving the *Galileo* spacecraft. Each change necessitated a complete redesign of the mission (i.e., the sequence of experiments and the schedule for ground station support). The Space Shuttle *Challenger* disaster in January 1986 delayed, then canceled, the 1986 launch date. Following the *Challenger* incident, NASA adopted a series of increased safety measures for the Space Shuttle program, one of which was the cancellation of the Centaur upper stage as a Space Shuttle payload. The Centaur upper stage was replaced by a low-risk, low-energy propulsion system called the inertial upper stage (IUS). Because the IUS did not have enough energy for a direct trajectory to Jupiter, *Galileo*’s trip to Jupiter was re-planned, for a fourth time, using a convoluted series of gravity assists, once from Venus and twice from Earth, which increased the journey to Jupiter from 27 months to six years. The new mission was launched in October 1989 and scheduled to reach Jupiter in December 1995. (A description of the IUS-to-Shuttle/orbiter telemetry link is given in [60]).

**Figure 13:**
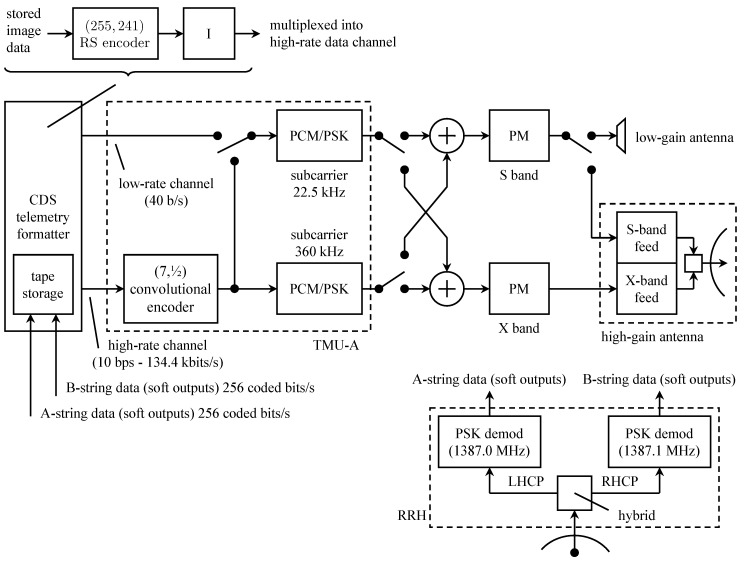
A block diagram of the originally planned Galileo orbiter telemetry system. Simplified from [61].

The originally planned telemetry system is summarized by the block diagram in Figure 13. The command data subsystem (CDS) is stored and formatted for transmission engineering and science measurements. The CDS produced two telemetry channels: a low-rate channel at 40 bits/s and a high-rate channel with an adjustable rate from 10 bits/s to 134.4 kbits/s. The use of the 40 bit/s CDS output was for safe-mode operation (when the onboard fault-protection software detects a problem that requires unplanned intervention via the command uplink). Within the CDS, image data were encoded with a (255,241) Reed–Solomon code and block interleaved with an interleaving depth of 4. The encoded image data formed part of the high-rate channel output from the CDS.

The two CDS outputs formed the inputs to the telemetry modulation unit (TMU). There were two TMUs, designated TMU-A and TMU-B. Initially, the two were identical. The low-rate channel data modulated the low-rate subcarrier. The high-rate channel data were encoded, using the (7,12) convolutional code developed for the *Voyager* program. The coded bits could modulate either the low-rate subcarrier or the high-rate subcarrier, depending on the selected data rate.

The TMU outputs (PCM/PSK-modulated subcarriers) could be applied to phase modulators at S-band (2300 MHz) or X-band (8400 MHz). The telemetry could be downlinked at the S-band alone, at the X-band alone, or both simultaneously. S-band transmission was via a low-gain antenna or a high-gain antenna. X-band transmission was possible only with the high-gain antenna.

The high-gain antenna was a 4.8-m parabolic reflector [61]. The reflector closed like an umbrella for storage, transport, and launch. The plan was to unfurl the antenna during the Earth-to-Jupiter segment of the voyage. The highest telemetry data rate (134.4 kbits/s) was only possible at the X-band with the high-gain antenna and the convolutional code.

The figure also shows the relay receiving hardware (RRH). This system was used to receive transmissions from the atmospheric probe. The probe transmitted two 128 bits/s streams in parallel at different frequencies and on different polarizations. The probe telemetry data were encoded using the same (7,12) convolutional code used by the TMU. The coded bit streams directly modulated L-band carriers at 1387.0 MHz (left-hand circular polarization) and 1387.1 MHz (right-hand circular polarization). The received signal was separated, synchronized, and matched filtered by the RRH. The soft-matched filter outputs (quantized to 3 bits) were stored for later transmission to Earth.

When the October 1989 launch date was announced, the impact on the mission was evaluated. The consequences of the delayed launch data and increased transmit time were profound. The December 1995 rendezvous occurred during the maximum Earth–Jupiter range and a southerly declination of $-23^{\circ }$ rather than the minimum Earth–Jupiter range with a northerly declination of $+18^{\circ }$ as originally planned. The maximum Earth–Jupiter range minimized the signal power at the receiving stations. The southerly declination meant most advantageous ground station sites were in the Southern Hemisphere, where there were fewer available ground stations. The output power of the Galileo power source, a radioisotope thermoelectric generator (RTG), decreased with time. By December 1997, the RTG was not capable of producing enough power to run the X-band transmitters at full power. Consequently, the X-band transmitters had to operate in low-power mode.

The combined effect was to produce received power levels well below the initially planned power levels. Without changes, the only way to close the link was to reduce the transmitted bit rate to such low rates that the viability of the science missions was threatened. In the two years leading up to the October 1989 launch, major upgrades to the capabilities of the ground stations and orbiters were pursued. The ground station improvements included upgrades to tracking systems, the development and installation of new RF components with lower noise figures, and a provision for arraying multiple ground stations [59].

For the orbiter upgrade, a new, more powerful, convolutional code was adopted, namely, the (15,14) code defined by the following polynomials:

(10) $$\begin{array}{cc} G^{\left(1\right)}\left(D\right) & =1+D^{3}+D^{4}+D^{7}+D^{8}+D^{10}+D^{14}\\ G^{\left(2\right)}\left(D\right) & =1+D^{2}+D^{5}+D^{7}+D^{9}+D^{10}+D^{11}+D^{14}\\ G^{\left(3\right)}\left(D\right) & =1+D+D^{4}+D^{5}+D^{5}+D^{7}+D^{9}+D^{10}+D^{12}+D^{13}+D^{14}\\ G^{\left(4\right)}\left(D\right) & =1+D+D^{2}+D^{6}+D^{8}+D^{10}+D^{11}+D^{12}+D^{14}. \end{array}$$

The design procedure that led to this choice is described in [62].

**Figure 14:**
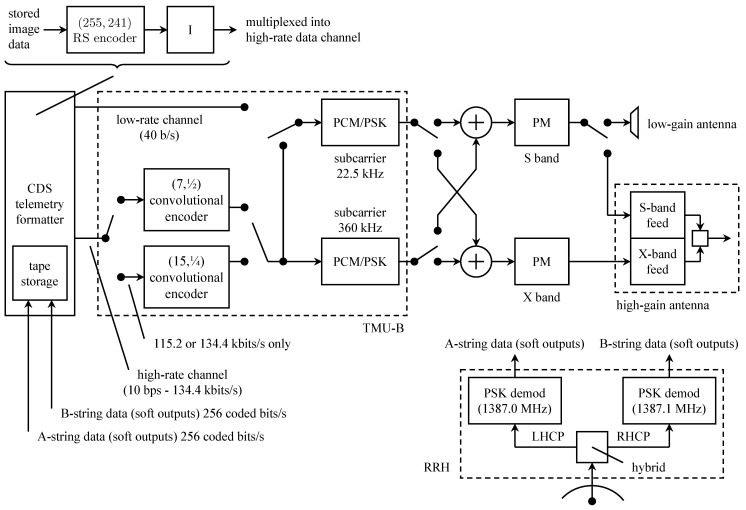
A block diagram of the modified Galileo orbiter system completed before launch. Only TMU-B is shown. TMU-A remained unchanged from its original configuration shown in Figure 13. Simplified from [61].

The hardware for the (15,14) convolutional encoder was installed in TMU-B. The encoder was designed for use with high-rate telemetry output at 115.2 or 134.4 kbits/s. The resulting orbiter configuration is shown in Figure 14. In the figure, only TMU-B is shown. TMU-A remained unchanged from its original configuration shown in Figure 13.

The addition of the longer convolutional on the orbiter initiated a hardware development effort to produce the corresponding decoder in the ground stations [63]. The decoder was a maximum-likelihood sequence detector using the Viterbi algorithm (VA) operating on a trellis with $2^{14}=16384$ states. The new decoder was dubbed the “Big Viterbi Decoder” (BVD) [64,65,66]. The BVD was programmable to decode any code with a constraint length from 2 to 15 and rates 1/2, 1/3, 1/4, 1/5, or 1/6 [63]. (The author worked at JPL during the summer of 1994 on a NASA/ASEE Summer Faculty Fellowship. His work area was in a laboratory where his desk was next to the BVD prototype. A manager (who shall remain unnamed) commented, “It is well-known JPL does software. It is well-known JPL does hardware. Now JPL does underwear.”, a pun on a popular product sold by the Fruit of the Loom company [67]).

Equipped with the addition of the hardware encoder in TMU-B, *Galileo* was launched in October 1989. In April of 1991, the command was sent to *Galileo* to unfurl the high-gain antenna. The antenna failed to unfurl properly [59,61]. This eliminated the use of high-gain antenna which, in turn, eliminated the use of X-band for the telemetry downlink. The only remaining communication link was through the low-gain antennas that operated only at the S-band. Once again, without changes, the telemetry downlink would have been limited to 10 b/s, severely compromising the scientific experiments that could be conducted. (The original plan, using the high-gain antenna at the X-band, could support 134.4 kbits/s).

The response was a series of changes, called the “*Galileo* S-band mission” that involved further upgrades to ground stations, advanced channel coding, and compression. The ground station upgrades included additional hardware upgrades and multi-site “arraying” [59] involving both inter-agency and international receiving sites. When receiving with only a single ground station antenna, the telemetry downlink was limited to about 10 bits/s. With arraying and improved channel coding (explained below), the telemetry downlink bit rate increased to the 40 bits/s–160 bits/s range.

**Figure 15:**
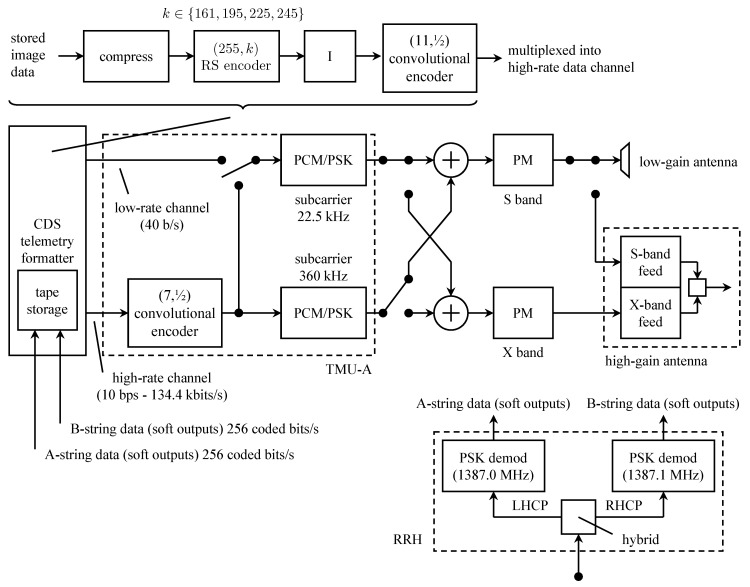
A block diagram of the modified Galileo orbiter system to support the “Galileo S-band mission”. Simplified from [61].

A new convolutional encoder, a modified Reed–Solomon encoder, and compression capabilities were added to the *Galileo* TMU. As before, images were encoded using concatenated coding using a length-255 Reed–Solomon outer code and a convolutional inner code. But the convolutional code, the interleaver, and the the Reed–Solomon code were changed. A simplified block diagram of the *Galileo* telemetry system is illustrated in Figure 15.

The outer code was the (255,k) Reed–Solomon code where *k* varied among four values in an 8-cycle pattern: k=161,245,225,245,195,245,225,245. The reasons for this are explained below. The field generator for the Reed–Solomon code is given by (8). The code generator polynomials are a generalization of (9):

(11) $$G\left(X\right)=\prod_{j=128-t}^{127+t}\left(X-\alpha^{11j}\right),\;t=\frac{n-k}{2}$$

where $\alpha^{11}$ is a primitive element in GF($2^{8}$). The codewords were interleaved with depth eight and convolutionally encoded. Because the (15,14) convolutional encoder in TMU-B could only operate at 115.2 or 134.4 kbits/s, it was unavailable to serve as the inner code for the Galileo S-band mission. Consequently, the inner code was changed to a (14,14) code. Convolutional encoding was accomplished using the cascade of a (11,12) convolutional encoder (in software, programmed into TMU-A) and the existing (7,12) convolutional encoder in the TMU hardware. The polynomials that define the composite (14,14) convolutional code are as follows [61]:

(12) $$\begin{array}{cc} G^{\left(1\right)}\left(D\right) & =1+D^{2}+D^{3}+D^{8}+D^{12}\\ G^{\left(2\right)}\left(D\right) & =1+D+D^{2}+D^{3}+D^{5}+D^{7}+D^{8}+D^{9}+D^{10}+D^{11}+D^{13}\\ G^{\left(3\right)}\left(D\right) & =1+D^{2}+D^{4}+D^{5}+D^{5}+D^{7}+D^{10}+D^{11}+D^{13}\\ G^{\left(4\right)}\left(D\right) & =D+D^{2}+D^{3}+D^{5}+D^{5}+D^{7}+D^{9}+D^{11}+D^{13}. \end{array}$$

Compression reduced the number of information bits to be stored on the orbiter and transmitted to Earth. This was an important development for making the transmission of image data viable. A description of the compression algorithm is given in [61].

At the ground station, an innovative decoding strategy was adopted. The decoding algorithm is described and analyzed in [68]. Decoding was accomplished in four stages.

Stage 1: The soft outputs of the PCM/PSK demodulator were applied convolutional decoder based on the VA. The VA outputs were deinterleaved. The first RS codeword, from the (255,161) RS code, was decoded. The (255,161) RS code, capable of correcting t=47 errors, is the most powerful of the four codes used for the outer code.
Stage 2: The detected symbols from Stage 1 were fed back to the VA for a second application of the VA assisted by the RS decoding decisions; called “redecoding” in [68]. The VA outputs were deinterleaved. The fifth RS codeword, from the (255,195) RS code, was decoded. The (255,195) RS code, capable of correcting t=30 errors, is the second most powerful of the four codes for the outer code.
Stage 3: The detected symbols from Stage 2 were fed back to the VA for a third application of the VA assisted by the previous RS decoding outputs. The VA outputs were deinterleaved. The third and seventh RS codewords, based on the (255,225) RS code, were decoded. The (255,225) code, capable of correcting t=15 errors, is the third most powerful of the four codes used for the outer code.
Stage 4: The detected symbols from Stage 3 were fed back to the VA for a fourth and final round of VA assisted by the previous RS decoding outputs. The VA outputs were deinterleaved. The second, fourth, sixth, and eighth RS codewords, based on the (255,245) RS code, were decoded. The (255,245) RS code, capable of correcting t=5 errors, is the least powerful of the four codes used for the outer code.

Despite all the failures, 70% of the mission objectives were met. The delayed launch date and extended voyage time created some science opportunities that would not have existed with the original plan. *Galileo* images two asteroids in transit to Jupiter: Gaspra (October 1991) and Ida (August 1993). The images of Ida showed it had a small moon, the first asteroid–moon system discovered. About 18 months before Galileo reached Jupiter, the spacecraft found itself uniquely positioned to image the impact of Comet Shoemaker-Levy 9 on the upper atmosphere of Jupiter in July 1994.

### 3.8. Cassini–Huygens

The *Cassini*–*Huygens* mission was an international collaboration involving the National Aeronautics and Space Administration (NASA), the European Space Agency (ESA), the Italian Space Agency (ASI), and European academic and industrial partners [69]. The mission was designed to explore the Saturnian system. The spacecraft comprised two parts, an orbiter called *Cassini* and a probe called *Huygens*. The probe separated from the orbiter and descended into the atmosphere of Titan (the largest Saturnian moon) eventually landing on Titan’s surface. The spacecraft was launched in October 1997 and was inserted into Saturn’s orbit in July 2004. The main mission lasted until 2007.

**Figure 16:**
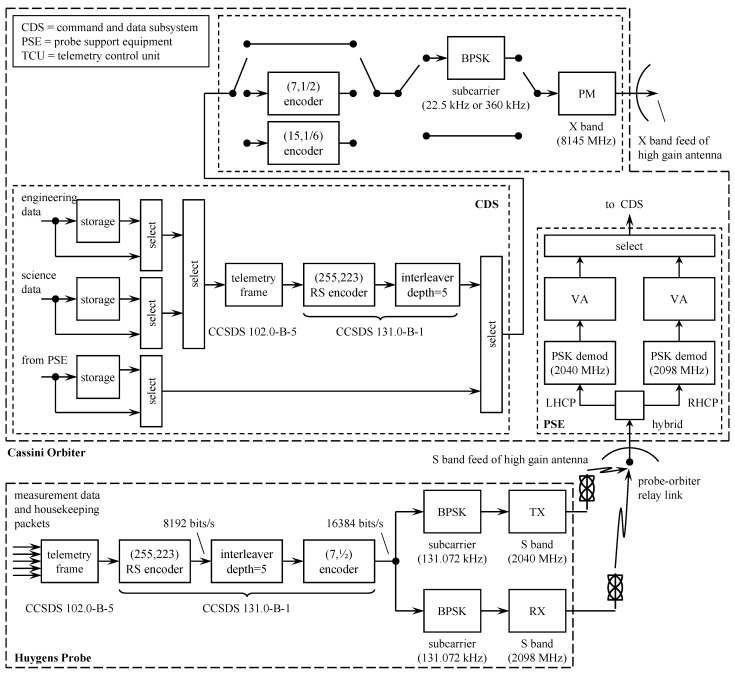
A simplified block diagram of the communication system and channel coding capabilities of the *Cassini*/*Huygens* space probe. Adapted from [69,73].

The orbiter-to-Earth telemetry system is illustrated in the top part of the block diagram in Figure 16. One of the functions of the command and data subsystem (CDS) was to collect engineering data, science data, and probe relay data. The collected data were either transmitted in real-time or stored for later transmission. Real-time engineering data were provided at a variable rate from 5 bits/s to 1869 bits/s. The science data rate varied, depending on the experiment, from 5 bits/s to 248,850 bits/s. The telemetry to be transmitted was framed following the CCSDS packet telemetry standard CCSDS 102.0-B-5 [70] and encoded using the same (255,223) Reed–Solomon code used by *Voyager* (see Section 3.5) [69]. Interleaving was performed by a depth-5 interleaver [69]. The framed, encoded, and interleaved packet produced by the CDS was transferred to the telemetry control unit (TCU) where it was optionally encoded with a (7,12) or (15,16) convolutional encoder. The optionally encoded data modulated a subcarrier or directly phase-modulated an X-band carrier. In the former case, the subcarrier-modulated signal phase-modulated the X-band carrier. The (7,12) convolutional code was the same one used by *Voyager* shown in Figure 12 and described by the polynomials (6). The (15,16) convolutional code is described by the following polynomials [65,71]:

(13) $$\begin{array}{cc} G^{\left(1\right)}\left(D\right) & =1+D^{3}+D^{4}+D^{7}+D^{8}+D^{10}+D^{14}\\ G^{\left(2\right)}\left(D\right) & =1+D^{2}+D^{5}+D^{7}+D^{9}+D^{10}+D^{11}+D^{14}\\ G^{\left(3\right)}\left(D\right) & =1+D+D^{2}+D^{6}+D^{8}+D^{10}+D^{11}+D^{12}+D^{14}\\ G^{\left(4\right)}\left(D\right) & =1+D+D^{4}+D^{5}+D^{6}+D^{7}+D^{9}+D^{10}+D^{12}+D^{13}+D^{14}\\ G^{\left(5\right)}\left(D\right) & =1+D+D^{2}+D^{4}+D^{5}+D^{7}+D^{9}+D^{10}+D^{11}+D^{12}+D^{13}+D^{14}\\ G^{\left(6\right)}\left(D\right) & =1+D+D^{2}+D^{3}+D^{4}+D^{6}+D^{8}+D^{11}+D^{13}+D^{14} \end{array}$$

and is characterized by the impressive $d_{\mathrm{free}}=56$.

The probe telemetry system is illustrated by the block diagram in the lower portion of Figure 16. Packets containing scientific measurement data and packets containing housekeeping data were used to form telemetry frames following the CCSDS packet telemetry standard CCSDS 102.0-B-5 [70]. The data were encoded using the same (255,223) Reed–Solomon code used by the orbiter. The Reed–Solomon encoded data were interleaved and encoded using the same (7,12) convolutional code used by the orbiter. The convolutional encoder output, at 16384 bits/s, BPSK modulated two subcarriers in parallel. Each subcarrier modulated a different S-band carrier. One of the S-band transmissions was delayed by about 6 s relative to the other to avoid data loss in the event a temporary link outage occurred, say, due to transmit antenna misalignment caused by strong winds in the Titan atmosphere [69,72]. On the orbiter, the parallel received signals were coherently detected and soft information was passed to the MLSE decoder, implemented using the VA, for the (7,12) convolutional code. The most reliable VA output was selected and passed to the CDS for storage and transmission to Earth. Note that Reed–Solomon decoding was *not applied* to the relay data. Consequently, the CDS did not have to apply the Reed–Solomon code to the data from the PSE. PSE data were applied directly to the selected convolutional encoder.

The *Huygens* probe was the first spacecraft to land on a body in the outer solar system. Prior to the landing, scientists were not certain that beneath the Titan’s opaque organic haze, there was a solid surface to land on [73]. Another interesting fact about the probe component of the mission was a problem called the “radio relay anomaly” [73,74]. Transmitter or receiver motion creates time compression (when moving toward each other) or time dilation (when moving away from each other). In most cases, the impact of the time compression/dilation on the underlying pulse train is negligible and is safely ignored. The resulting time-varying phase causes a frequency shift in the RF carrier known as a Doppler shift. What was overlooked during the design stage was the fact that the relative velocity of the orbiter as it flew past Titan while the probe descended was so large that the time compression/dilation could not be ignored. The compression/dilation was enough to cause cycle slips in the timing PLLs. The cycle slips would have caused large quantities of data to be lost. The obvious compensation, increasing the bandwidth of the timing PLL in the PSK demodulators in the orbiter’s PSE, was not possible because the timing PLL loop bandwidth was fixed in hardware. An innovative solution was developed by the *Huygens* Recovery Task Force: change the profile of the *Cassini* fly-by to reduce the relative velocity between the orbiter and the probe [73,74]. An interesting story on the ESA engineer who identified this problem, Boris Smeds, was published in [75]. The radio relay anomaly illustrates an important characteristic of channel coding that can be easily overlooked: many non-coding things have to work well before performance gains of coding can be realized.

### 3.9. In Search of the Missing 2 dB—Turbo Codes

Turbo codes were introduced in 1993 [76] and quickly became the subject of intense investigation in academia and industry. The space telemetry community also contributed to the subsequent research on turbo codes.

At the time turbo codes were introduced, the concatenated coding scheme involving the (255,223) Reed–Solomon code, interleaving, and the (7,12) convolutional code was the standard. This was the channel code first used in *Voyager*: see Figure 11c in Section 3.5. For years, there had been an effort to find channel codes whose performance was closer to the Shannon limit [71]. The first step toward this goal was the (15,1/6) convolutional code used by *Cassini*. The generator polynomials are given by (13). The decoder used the Viterbi algorithm operating on a trellis with $2^{14}=16384$ states. Turbo codes presented the opportunity to achieve the goal with lower complexity.

The U.S. space program “tiptoed” into the modern coding era [77] with the iterative decoding procedure used with the *Galileo* program described in Section 3.7. However, the *Galileo* decoder was the solution to the unusual set of circumstances that challenged the success of the *Galileo* mission. It was not seen as a general, long-term solution to moving closer to the Shannon capacity.

Thus, turbo codes presented a structured way to move closer to Shannon capacity with manageable decoder complexity. The hunt was on to establish a turbo coding standard for space telemetry. Divsalar and Pollara found turbo codes that minimized the decoded probability of *bit* error in 1995 [78]. In the EU, it was realized that because telemetry comprises data frames, the probability of *frame* error is more important than the probability of *bit* error [79]. To this end, turbo codes that minimized the probability of frame error were found and published by Calzolari and his team in 1998 [80]. The turbo codes in [80] were adopted by the CCSDS the TM Synchronization and Channel Coding Standard [50], less than six years after the introduction of turbo codes by Berrou in 1993.

The turbo code standardization process moved quickly because (1) few proposals were involved, and (2) turbo codes are defined by a relatively small number of parameters (e.g., rate, constraint length, register connections) [77]. The CCSDS turbo code comprises the parallel concatenation of two identical rate-1/4 systematic convolutional codes. The code is capable of producing turbo codes at rates of 1/2, 1/3, 1/4, and 1/6. The constituent convolutional codes had 16 states, in contrast to the 8-state codes standardized for 3G cellular systems and the IEEE 802.16 standard [77]. This choice was made because the constituent 16-state convolutional codes produced a turbo code with a fraction of dB more coding gain than the 8-state convolutional codes. In space telemetry, an improvement in coding gain at the expense of a factor of 2 in decoding complexity is generally a desirable trade-off.

**Figure 17:**
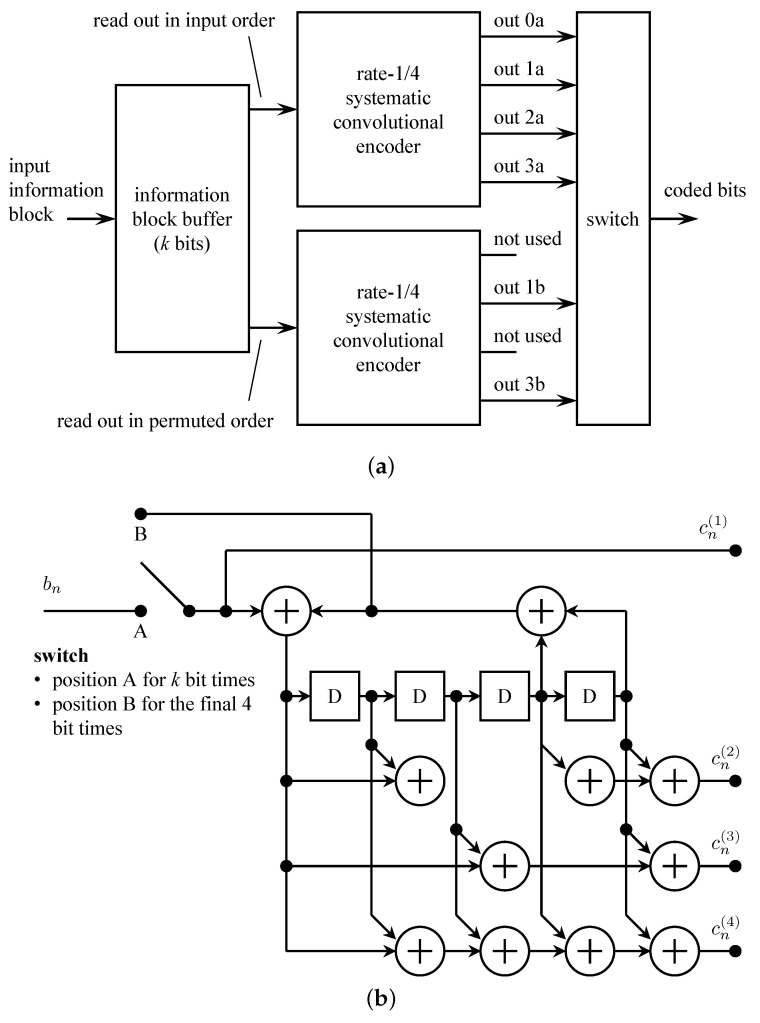
The CCSDS turbo code encoder: (**a**) The turbo code structure based on two identical rate-1/4 systematic convolutional encoders and an information block buffer that also serves as an interleaver; (**b**) A block diagram of the constituent rate-1/4 systematic convolutional encoder.

The CCSDS turbo encoder is illustrated in Figure 17. The arrangement producing parallel concatenation of the constituent codes is shown in Figure 17a. The input information block, comprising k∈{1784,3568,7136,8920} information bits, is stored in an information block buffer. The possible values of *k* correspond to Reed–Solomon interleaving depths of 1, 2, 4, and 5, respectively. The buffer also serves as the interleaver; interleaving is performed by reading out the *k* information bits in a permuted order specified in Section 6.3 g of [50]. The turbo code rate is determined by the connections between the outputs of the two constituent encoders and the overall output. The relationship is defined in Table 1.

The rate-1/4 systematic convolutional encoder is shown in Figure 17b. The switch is in position A for the first *k* input bits. The switch then moves to position B for 4 bits for trellis termination. Trellis termination refers to the state of the encoder at the end of transmission. The performance of the decoder (the *sequence* estimator) is improved when the beginning state and the ending state of the encoder are known. For non-recursive encoders, trellis termination is straightforward: append zeros to the end of the information block to produce the all-zeros state in the encoder. For recursive encoders (the case here), the bits to be appended depend on the encoder state after the input of the *k*-th information bit. Divsalar–Pollara [81] showed that a feedback method can be used to terminate the encoder in the all-zeros state. The Divsalar–Pollara technique is the one used in the CCSDS standard.

The first use of turbo codes in space was part of an experimental package on ESA’s *Small Missions for Advanced Research in Technology*-1 (*SMART*-1) that was launched to the Moon in September 2003. An experimental instrument called KaTE (Ka-band transmission experiment) carried a CCSDS turbo encoder. The KaTE instrument transmitted rate-1/4 turbo-encoded frames that were successfully received at ESA’s tracking site at Villafranca del Castillo, Spain, and decoded by a turbo decoder developed by Space Engineering Rome (Italy) and Politecnico of Torino, Italy on behalf of the European Space Agency [82,83].

As an experiment, turbo-coded transmissions from *SMART*-1 were not used for a significant telemetry downlink [77]. The first mission to use CCSDS turbo codes in the main telemetry downlink was NASA’s *Messenger*, the first Mercury orbiter, launched on August 2004 attaining Mercury orbit in March 2011. *Messenger* used the CCSDS rate-1/6 turbo code with k=8920 to encode telemetry at a variety of rates up to 104 kbits/second [82]. The main telemetry downlink was in the 8.4 GHz band [84] using the first phased array antenna deployed in space [85,86].

ESA’s *Rosetta* spacecraft was launched in March 2004 and attained orbit around the comet 67P/Churyumov–Gerasimenko in August 2014. *Rosetta* was the first spacecraft to *orbit* a comet. A lander called *Philae* detached from the orbiter *Rosetta* and performed the first successful landing on a comet in November 2014.

The *Rosetta*-to-Earth telemetry link operated at 5-20 kbits/s, depending on the distance [87]. The original plan was to use the NASA/CCSDS standard concatenated code comprising the (7,1/2) convolutional code as the inner code, an interleaver, and the (255,233) Reed–Solomon code as the outer code. The CSSDS turbo encoder was programmed into the software chain feeding the PM modulator [79]. When the CCSDS turbo code was used, the concatenated code was bypassed.

The *Philae*–*Rosetta* RF telemetry link operated at 2033.2 MHz using QPSK with the square-root raised-cosine pulse shape [88]. The data rate was 16.384 kbits/s. The CCSDS standard (7,1/2) convolutional code was used on the link. Soft-decision decoding using the Viterbi algorithm was performed on *Rosetta*.

The experiments using the CCSDS turbo codes were so successful that CSSDS turbo codes have been used in most subsequent missions by both NASA and ESA [89]. A particularly noteworthy program is *New Horizons*, launched in January 2006 to explore the Pluto-Charon system in the Kuiper belt. The Pluto encounter occurred in July 2015 [90]. After the Pluto encounter, the *New Horizons* mission was extended to study a Kuiper belt object then known as (486958) 2014 MU_69_, now called Arrokoth. The Arrokoth encounter occurred in January 2019.

**Figure 18:**
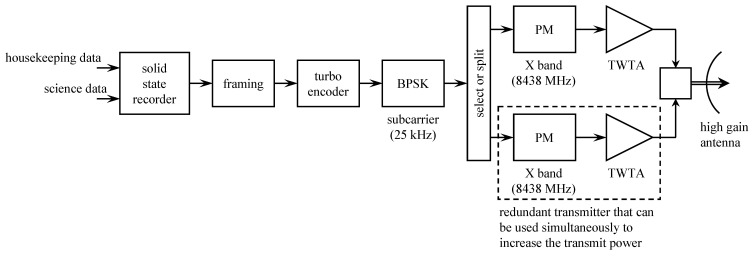
A block diagram of the *New Horizons* telemetry system.

A block diagram of the *New Horizons* telemetry system is illustrated in Figure 18. A real-time telemetry data link was not possible during an encounter. During an encounter, the spacecraft was positioned for scientific data collection. When the spacecraft was positioned for scientific data collection, the high-gain antenna was usually not pointed to Earth. Consequently, scientific measurements were stored in onboard solid-state memory. The data were encoded using the CCSDS rate-1/6 turbo code [91]. The encoded data modulated a 25 kHz subcarrier [91]. The subcarrier phase-modulated an X-band carrier [92] to produce a coded PCM/BPSK/PM signal. At Pluto, a bit rate of about 1 kbits/s was possible using one of the two redundant 12-W TWTAs. Using both TWTAs, the bit rate increased to 2 kbits/s [93]. The encoder used for the telemetry link is the rate-1/6 turbo code using k=1748 bits.

During the Pluto encounter, *New Horizons* collected about 6.25 GB of data [94]. The dataset was transmitted to Earth after the encounter at an average rate of 2 kbits/s using both TWTAs. The downlink took 15 months and was completed at the end of October 2016 [90]. The Arrokoth encounter took place in January 2019 and it took 20 months to downlink all the data at about 1 kbit/s. *New Horizons* was the first to image Pluto and other Kuiper belt objects.

### 3.10. In Search of the Missing 2 dB—Low-Density Parity Check (LDPC) Codes

In contrast to the fast and efficient process for developing a turbo code standard, the development of an LDPC coding standard for CCSDS has been more difficult. This is because the standards process faces a number of important challenges. First, because LDPC codes are defined by a sparse parity check matrix with large dimensions, there are a large number of possible codes. Second, if no structure other than sparseness is imposed on the code design, the code cannot be described by a small number of parameters whose values can be optimized in the way it is done for turbo codes. Third, there is a long list of desirable attributes [95], and no LDPC code is superior in all categories [77].

The CCSDS standards process considered the irregular repeat-accumulate (IRA) LDPC code in the second generation Digital Video Broadcast standard (DVB-S2) [96], other irregular LDPC codes, and regular LDPC codes [77]. In August 2011, CCSDS adopted a family of nine LDPC codes proposed by JPL in the 131 Blue Book [50].

The CCSDS LDPC codes are derived from a family of codes known as Accumulate Repeat-4 Jagged Accumulate (AR4JA) where the name derives from the protograph structure used to define the code [77]. The nine LDPC codes are binary (n,k) block codes. The family comprises two values of *k* and three values for *n* for each value of *k*. The values are summarized in Table 2. The interesting feature of these codes is that the rate is changed by changing the code length *n* rather than its dimension *k*. This provision was made to make the interface to telemetry packets transparent to the code rate.

An interesting application of CCSDS LDPC codes is the Mars Relay Network. The Mars Relay Network comprises a series of satellites in orbit around Mars (as of 2018, there were five operational options [97]) that receive data from “rovers” on the Martian surface and re-transmit that data to Earth [98]. The Mars Science Laboratory (or *Curiosity* rover) has the capability to transmit its measurements directly to Earth or via the Mars Relay Network. Direct transmissions to Earth use the CCSDS rate-1/6 turbo code and an X-band carrier [99]. The Mars-to-orbiter portion of the Mars Relay Network uses LDPC codes [100,101] using the CCSDS Proximity-1 space link protocol [102,103] and a UHF carrier. The majority of the data transfer from Martian “rovers” to Earth occurs via the Mars Relay Network [98,104].

### 3.11. Modern Codes in Future Deep-Space Telemetry

Fountain codes grew out of the data network world where communication over a binary erasure channel with unknown or quickly changing erasure probabilities was of interest [105]. For a set of *k* input symbols, a fountain code produces a potentially limitless set of output symbols where each output symbol is the sum of a subset of the input symbols where subset membership is defined by a distribution. The decoder produces the original set of *k* symbols with high probability from an observation comprising *n* symbols. Because *n* is not fixed, the fountain code is a rateless code. The closer *n* is to *k*, the better the fountain code. The first practical rateless codes were introduced by Luby [106] and called LT codes. Raptor codes are constructed on LT codes by applying a precoder in the form of an outer code [105]. The ability of a rateless code to operate in a variety of channels without a fixed rate has made them of interest in deep-space communications. An interesting example is the study [100] where a joint source coder and channel code, based on Raptor codes over GF(4), were applied to images from the Mars Exploration Rover. The new source/channel coding approach was shown to have advantages over the adaptive rate scheme based on the CCSDS LDPC codes used for the Mars Relay Network.

A polar code is another form of a rateless code introduced by Arikan [107]. Polar codes have recently been adopted as the channel code for the uplink and downlink control information channel in the 5G enhanced mobile broadband (eMBB) service [108]. The absence of “error floors”, characteristic of LDPC bit error rate performance, together with relatively low complexity decoding algorithms, makes polar codes an attractive option for deep-space communications. A recent study [109] compared polar codes to the CCSDS LDPC codes for deep space optical links. The results were mixed, depending on the assumptions adopted for the simulation, but showed that for short block lengths, polar codes outperformed the LDPC codes.

Channel coding with short block length codes has been of recent interest in machine-type communications, the Internet of Things, and command links, such as those used for deep-space communications [110]. Coding using short block length codes in deep-space communications was examined in [111]. Here, the common approach of including CRC bits to a block of information bits prior to convolutional encoding is reexamined for the case of short block lengths. A list Viterbi algorithm was developed based on the view of a concatenated coding system. The results showed a nearly 3-dB improvement in coding gain relative to the Viterbi algorithm operating only on the convolutional code.

## 4. Aeronautical Mobile Telemetry

### 4.1. Channel Coding in the 20th Century

From 1966 to 1999, 245 papers mentioning what is now called channel coding appeared in the *Proceedings of the International Telemetering Conference*. The treatment of coding ranged from incidental remarks that channel coding improves link reliability to full-length treatments of code design and decoding algorithms. The most common mention of coding was the discussion of ground stations, for both satellites and space probes, that had decoding capabilities conforming to CCSDS coding standards [50]. It is clear that by the 1970s coding became permanently established in NASA circles, but was little more than “in the air” in aeronautical mobile telemetry circles.

Of the 245 papers, only 40 target aeronautical mobile telemetry systems: ref. [112,113,114,115,116,117,118,119,120,121] mention the use of coding in magnetic tape recording, ref. [122,123,124] mention the use of coding in solid state recording, ref. [125] describes the use of coded ATM technology for ground transport, ref. [126] predicts the use of channel codes for improving quality telemetry data transport on a data bus inside a Boeing aircraft, ref. [127] describes how coding might be used to assess the quality of merged PCM streams from different receivers, and [14,128,129,130,131,132,133,134,135,136,137,138,139,140,141,142,143,144,145,146,147,148,149,150] deal specifically with the air-to-ground radio link.

In a 1975 “where do we go from here” paper, Reynolds states [151], “error-detecting- and correcting codes will receive much more attention”. It is interesting that Strock, in two “future trends” papers published in 1978 [10] and 1988 [152], does not mention channel coding. This is because, in the era in which he wrote, the reliability of RF transmission was not the bottleneck. The bottlenecks were recording bandwidth, ground station configuration, and the ability of data processors to produce real-time displays of telemetry data with the proper engineering units. The same is true for Rauch’s “Telemetry Systems of the Future” published in 1989 [153]. Van Doren’s “Airborne Telemetry Trends for the 1990’s” [154] states (rather briefly), “convolutional encoding enhances link performance”, in the context of future breakthroughs in FM. A decade later, Yates’ Future Trends paper [155] fails to mention coding. None of these observations are intended as criticisms. Instead, these observations reflect the reality of the times in which these papers were written. Other issues were more pressing, such as spectral congestion, the ability to automatically organize hundreds of thousands of measurements, store those measurements, and use networks to convey those measurements to the engineers and scientists who need them.

The first coding study published in the open literature constrained by the aeronautical mobile telemetry environment was carried out by Gardner and Levy [128] in 1983. They examined BPSK with the (7,12) convolutional code whose encoder is shown in Figure 12 and described by the polynomials (6). Interleaving was applied after the encoder and the corresponding deinterleaver prior to the Viterbi decoder to “break up” the long error bursts caused by link outages. The decoder was able to support a 10 Mbit/s downlink. Except for the placement of the interleaver and deinterleaver, this system was the inner code for the *Voyager* program described in Section 3.5. To the author’s knowledge, this configuration was never flown on a test range. The most probable reason is that it required too much bandwidth.

**Figure 19:**
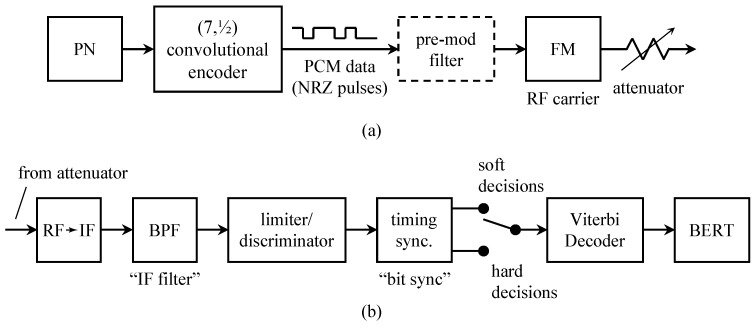
The experimental configuration for the coded PCM/FM experiment described in [129]: (**a**) the encoder and transmitter; (**b**) the demodulator and decoder.

The next serious investigation, also performed in 1983 by Cox and Nichols [129], addressed the bandwidth issue by exploring the same rate-1/2 constraint length 7 convolutional code with PCM/FM. The experimental configuration is shown in Figure 19. A pseudo-noise (PN) sequence was encoded and input to an FM transmitter. No pre-modulation filter was used in this experiment. The value of $E_{b}/N_{0}$ was set by a variable attenuator (the thermal noise source was the noise generated by the receiver). The bit error rate performance, measured by the bit error rate tester (BERT) operating on a local copy of the PN sequence, was quantified experimentally for both soft decision decoding and hard decision decoding and for different IF filter bandwidths. Soft decision decoding showed little to no improvement over hard decision decoding. This was explained by the fact that the limiter/discriminator output is not conditionally normal, at low signal-to-noise ratios, and displays well-known “pop noise” that the soft decision metrics are not equipped to handle. The decoded bit error rate improved as the IF bandwidth increased. This is explained by the fact that inter-symbol interference—something the decoder is not designed for—diminishes as IF bandwidth increases. Coding gain was limited to 2.2 dB. Had a pre-modulation filter been used, the inter-symbol interference would have been more severe thus reducing the coding gain even further. In the end, it was not felt that coded PCM/FM, in the form examined in [129], was a practical solution for improving link reliability. Coded PCM/FM systems with good performance were still 40 years in the future.

**Figure 20:**
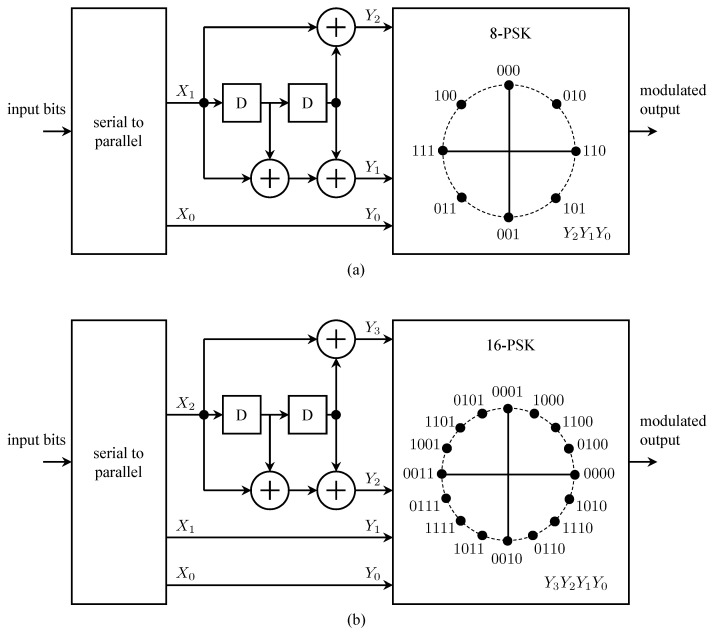
Trellis-coded modulation encoder/modulator studied by Carden, Kopp, and Ross [130,131,133,134]: (**a**) the 4-state trellis code mapping to 8-PSK; (**b**) the 4-state trellis code mapping to 16-PSK.

In the late 1980s, to address the undesirable bandwidth expansion accompanying channel codes, Carden, Kopp, and Ross [130,131,133,134] investigated the application of trellis-coded modulation (TCM) [156,157,158,159] to aeronautical mobile telemetry. Trellis-coded modulation is a form of coded modulation well suited to bandwidth-constrained applications. For a fixed information bit rate, the signal set is expanded to account for the code rate; the sequence of symbols in the expanded signal set is restricted by the code to maximize the Euclidean distance between allowed sequences. The encoder/modulator is illustrated in Figure 20. Two versions were studied: coded modulation for 8-PSK shown in Figure 20a and coded modulation for 16-PSK shown in Figure 20b. Both modulations were based on the same rate-1/2 4-state trellis code shown in the figures. The signal set was restricted to PSK to create a modulated carrier with a constant envelope—required because the RF power amplifiers operated in full saturation. The corresponding detector comprised the Viterbi algorithm operating on the matched filter outputs with the 4-state trellis. Quantized versions of the matched filter outputs were studied [130,133,134,160,161]. The simulation results with quantization showed that the 8-PSK system achieved a 1.5 dB improvement over the QPSK and that the 16-PSK system achieved a 0.5–1.0 dB improvement over QPSK. The limited improvements were due to quantization, the use of a 4-state code, and the PSK constraint.

The TCM system was originally developed under a NASA grant for the space station flight telerobotic servicer [131]. It was certainly considered by the aeronautical mobile telemetry community but was probably not given serious consideration due to limited performance gains relative to the increase in complexity. To the author’s knowledge, this system was never flown on a test range.

**Figure 21:**
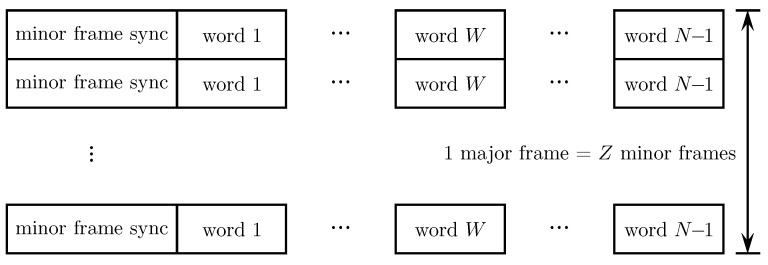
A diagram of a simple PCM format comprising minor frames of *N* words and a major frame of *Z* minor frames [cf., Figure 2a].

The first “close call” using channel coding was the Bell Boeing V-22 Osprey [162] tests conducted at the Naval Air Station (NAS), the Patuxent River, MD, USA, in the late 1990s. The fundamentals of PCM telemetry are required to understand the rationale behind the coding strategy. PCM telemetry transmits *framed* data continuously. *Frame* refers to the periodic insertion of markers, such as major frame and minor frame markers, to divide the data into frames even though transmission is continuous. A very simple PCM frame structure is illustrated in Figure 21. One round through the commutator connected to N−1 sensors produces N−1 data words. [See Figure 2a] Each data word is 4–32 bits in length. The N−1 data words define a minor frame. The start of a minor frame is marked by a minor frame synchronization word, 16–33 bits in length. (Major frames are usually identified by a subframe counter known as a subframe ID in Chapter 4 of IRIG 106 [163]. The subframe ID counts 1 to *Z*, then starts over at 1; the “1” indicates the start of a major frame. When used the subframe ID occupies the position of word 1 in Figure 21). Counting the minor frame synchronization word, each minor frame comprises *N* words and is represented by a row in Figure 21. *Z* minor frames define a major frame. In this simple example, the *W*-th word in each minor frame is from the same sensor; the *W*-th column in Figure 21 comprises consecutive samples from the same sensor. The PCM frame structure is more complex when sub-commutation or super-commutation is used. See Chapter 4 of [163].

In the V-22 tests, the test article instrumentation included hardware to compute CRC parity bits (eight bits) for error detection on each PCM minor frame. The check bits occupied the position of Word 1 in Figure 21. The ground station was equipped with a bypassable hardware parity check unit called the “frame thrower” [164]. Minor frames that did not pass the parity check were discarded and not passed on for recording or display.

The assessment of error detection was visual: strip chart plots of measurements were used, where errored minor frames were discarded to show smooth curves. This is because most sensor outputs were oversampled so the elimination of occasional samples did not negatively impact the reconstructed continuous-time measurements [164]. In contrast, the retention of errored frames produced strip chart plots with spikes due to the errors.

Despite the advantages of error detection, the project managers did not approve the use of error detection during actual flight tests. The fear was that discarding minor frames with a failed parity check would lead to missing data. The customer preferred to see unusable data due to dropouts instead of clean data [164].

As of this writing, parity check bits on minor frames is an option in the IRIG 106 standards [163]. The standard recognizes two 16-bit options and one 32-bit option. The two 16-bit options are defined by the polynomials

(14) $$\begin{array}{cc} \mathrm{CRC}\text{-}16\text{-}\mathrm{ANSI}: & \;X^{16}+X^{15}+X^{2}+1\\ \mathrm{CRC}\text{-}16\text{-}\mathrm{CCITT}: & \;X^{16}+X^{12}+X^{5}+1. \end{array}$$

The 32-bit option is defined by the polynomial CRC-32 given by

(15) $$X^{32}+X^{26}+X^{23}+X^{22}+X^{16}+X^{12}+X^{11}+X^{10}+X^{8}+X^{7}+X^{5}+X^{4}+X^{2}+X+1.$$

The length-32 CRC is defined in the IEEE 802.3 standard [165]. When used, the 16 or 32 parity check bits are positioned at the end of the minor frame.

By the late 1990s, the bandwidth issue became an acute problem. The causes were mentioned in Section 2: increasing test article complexity increased bit rate requirements and federal spectrum auctions reduced the spectrum available for aeronautical mobile telemetry [16]. Combined, these trends reduced the spectrum available to aeronautical mobile telemetry at the same time the demand for spectrum was increasing. At the same time, the increasing complexity of test articles also exacerbated a problem that previously had been more of a secondary problem: data reliability. More complex test articles tend to have a wider operating range. The wider operating range increases the distance over which the telemetry link must be maintained. This, coupled with increased bit rates, reduced the received value of $E_{b}/N_{0}$.

The response was a series of initiatives funded by the advanced-range telemetry (ARTM) program [144,145]. Of the many ARTM goals, two are relevant to the focus of this article: bandwidth-efficient modulation and channel coding. Modulations with improved spectral efficiencies were adopted in two steps or “tiers”. First were the modulations with twice the spectral efficiency of PCM/FM but with the same detection efficiency as PCM/FM using limiter/discriminator detection: Feher’s patented QPSK version B (FQPSK-B) and shaped offset QPSK version TG (SOQPSK-TG). FQPSK-B is a proprietary version of FQPSK [166,167,168,169] with a quasi-constant envelope. It was adopted in the IRIG 106 standard in 2000 [170]. An improved version of FQPSK-B, called FQPSK-JR [171], was closer to constant envelope and was adopted in the IRIG 106 standard in 2004 [172]. SOQPSK was described in 2000 by Hill [173] and was truly a constant envelope. Hill introduced two versions called SOQPSK-A and SOQPSK-B. SOQPSK-A had a slightly narrower bandwidth and worse detection efficiency than SOQPSK-B. In 2004, the Telemetry Group (TG) of the Range Commanders Council adopted a compromise version called SOQPSK-TG in the IRIG 106 standard [172]. SOQPSK-TG was a fully interoperable alternative to FQPSK-B [173,174].

The second phase or “tier” identified modulations with three times the spectral efficiency of PCM/FM but with the same detection efficiency as PCM/FM using limiter/discriminator detection. The result was a non-binary partial response two-index continuous phase modulation (CPM) called ARTM CPM. ARTM CPM was adopted by the IRIG 106 standard in 2004 [172].

An important feature of the new modulations is that all are *digital* modulations. Consequently, they are well-positioned to incorporate channel coding. As of this writing, PCM/FM, SOQPSK-TG, and ARTM CPM are included in the current telemetry standards IRIG 106-23 [163]. Despite the name of the second one, all three are variations of CPM [175,176,177]. The search for new modulations, at the time, was restricted to CPMs because they have constant envelopes. Constant amplitude signals are well-matched to RF power amplifiers operating in their most power-efficient mode: full saturation. These modulations formed the basis for the exploration of channel codes in the 21st century.

### 4.2. Channel Coding in the 21st Century

The first serious test of channel coding with error correction capabilities in a realistic aeronautical mobile telemetry environment was described by Rymer [14]. In the tests, a provision was made to store data on the test article. The same data were transmitted to the ground station and the data bit decisions were recorded on the ground. Error patterns were generated by comparing the two. The datasets used to generate the error patterns were derived from tests involving the T-39 Sabreliner [178] at Edwards AFB, the F/A-18 Hornet [179] at NAS Patuxent River, and the V-22 Osprey [162] also at NAS Patuxent River [14]. As a result, the error patterns captured the realities of the aeronautical mobile telemetry channel: a combination of random errors and burst errors, the latter due to signal dropouts caused by difficult propagation scenarios. The experiment tested the performance of an interleaver followed by a (255,k) Reed–Solomon code for k= 224, 207, 199, and 146. The test results were mixed. The coding technique performed well as long as the deinterleaved error bursts did not overwhelm the error-correcting capability of the code. Error bursts lasting longer than 500 ms were a problem. Unfortunately, many of the dropouts were 500 ms or more. The findings were consistent with earlier simulations using length-255 Reed–Solomon codes reported in [148] based on error sequences derived from F-16 flight data described in [147,180].

Turbo codes were introduced by Berrou et al, in 1993 [76]. The original code was based on a parallel concatenation of recursive convolutional codes. Convolutional codes work well for low-rate coding applications. In aeronautical mobile telemetry, where severe bandwidth constraints are dominant, the bandwidth expansion associated with low-rate codes renders them impractical. Motivated by the trend that block codes tend to perform better for high-rate coding applications, Pyndiah introduced turbo product codes in 1994 [181]. By 2002, at least one single-chip solution was available on the commercial market [182].

**Figure 22:**
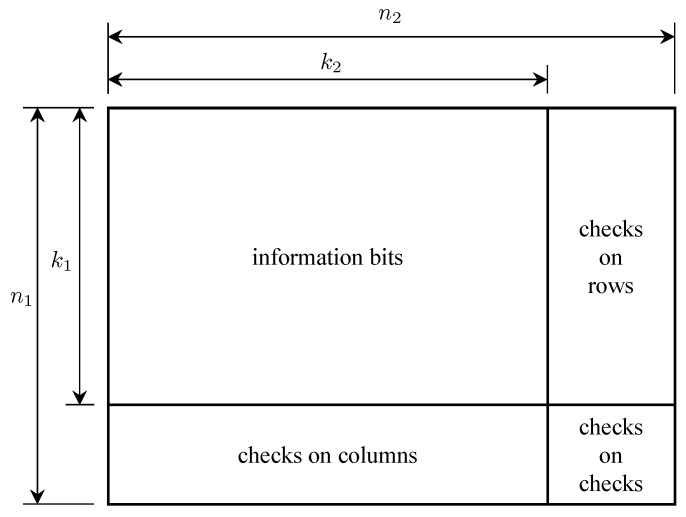
A graphical representation of encoding used by a turbo product code based on $\left(n_{1},k_{1}\right)$ and $\left(n_{2},k_{2}\right)$ linear block codes.

A product code is based on the “product” of an $\left(n_{1},k_{1}\right)$ linear systematic block code and an $\left(n_{2},k_{2}\right)$ linear systematic block code. The resulting code is usually denoted as an $\left(n_{1},k_{1}\right)\times \left(n_{2},k_{2}\right)$ code. A graphical representation of the encoder is shown in Figure 22 [181]. A block of $k_{1}\times k_{2}$ information bits is read into a $k_{1}\times k_{2}$ array. The $\left(n_{2},k_{2}\right)$ code is applied to each row of the block to produce an intermediate block of $k_{1}\times n_{2}$ bits. The $\left(n_{1},k_{1}\right)$ code is applied to each column of the intermediate block to produce an $n_{1}\times n_{2}$ block of coded bits. The bits are read out in a predefined fashion (e.g., row-by-row, column-by-column, or along diagonals) and used to modulate an RF carrier. The code rate of the product code is $k_{1}k_{2}/\left(n_{1}n_{2}\right)$. At the decoder, soft information from the demodulator is organized into an $n_{1}\times n_{2}$ matrix corresponding to that in Figure 22. Soft decision decoding alternates between the rows and columns in an iterative fashion using the turbo principle [181].

Laboratory experiments using PCM/FM and SOQPSK-TG are described in [183]. Soft output information for PCM/FM was derived from the trellis detector. Soft output information for SOQPSK-TG was derived from the detection filter outputs. (See [184] for a description of the SOQPSK-TG symbol-by-symbol detector and its application to soft-decision decoding). The experiment evaluated the highest rate codes produced by the AHA4540 encoder/decoder chip: the (32,26)×(32,26), (64,57)×(64,57), and (128,120)×(128,120) product codes. The measured coding gains were 3.0–3.5 dB for PCM/FM and 6 dB for SOQPSK-TG. (Note that the coding gain for turbo-product coded PCM/FM with trellis detection relative to uncoded PCM/FM with limiter/discriminator detection is 3 dB better: 6–6.5 dB).

Flight tests using the (32,26)×(32,26) turbo product code were reported in [185]. The measured coding gain was 3 dB for PCM/FM and 4-5 dB for SOQPSK-TG. Because the bit error rate vs. $E_{b}/N_{0}$ curve is steep for coded links, it was observed that the coded link was either error-free or lost. It was noted the resynchronization time (in the wake of a link outage) was much greater for the coded system than for the uncoded system, but that the increased resynchronization time did not have much of an impact on the overall link availability. This is because bit errors and link outage/resynchronization reduce link availability in the uncoded link. In contrast, only link outage/resynchronization reduces link availability in the coded link. The recommendation was positive: the cost of bandwidth increase (256/169≈1.5) was justified by increased reliability. The (64,57)×(64,57) was seriously considered by Boeing for use in testing the Boeing EA-18G Growler [186] at NAS Pax River [187,188]. In the end turbo product codes were not used for the EA-18G tests due to concerns over long resynchronization times [189]. Turbo product code development was dropped and disappeared into obscurity in aeronautical mobile telemetry circles.

The next set of efforts, described in Section 4.2.1–Section 4.2.4, have been field-tested, are currently in use, or are actively being developed for future use.

#### 4.2.1. Enhanced Flight Termination System (EFTS)

To prevent unmanned test articles from leaving the controlled airspace of a test range, unmanned test articles are equipped with a flight termination system (FTS). An FTS comprises two elements: a ground-based transmitter that uplinks commands to the unmanned test article, and a flight termination receiver installed on the unmanned test article that receives the uplink commands. When an unmanned test article veers off the range, a range safety officer issues a “terminate” command that “halt[s] the forward motion of an errant vehicle by placing it in a condition of zero lift (nominally) and zero thrust to establish a known impact point or debris footprint”. [190]. The mechanism for termination varies and includes zero thrust with a parachute, a flat spin, or destruction by an explosive charge (usually for a missile or rocket).

**Figure 23:**
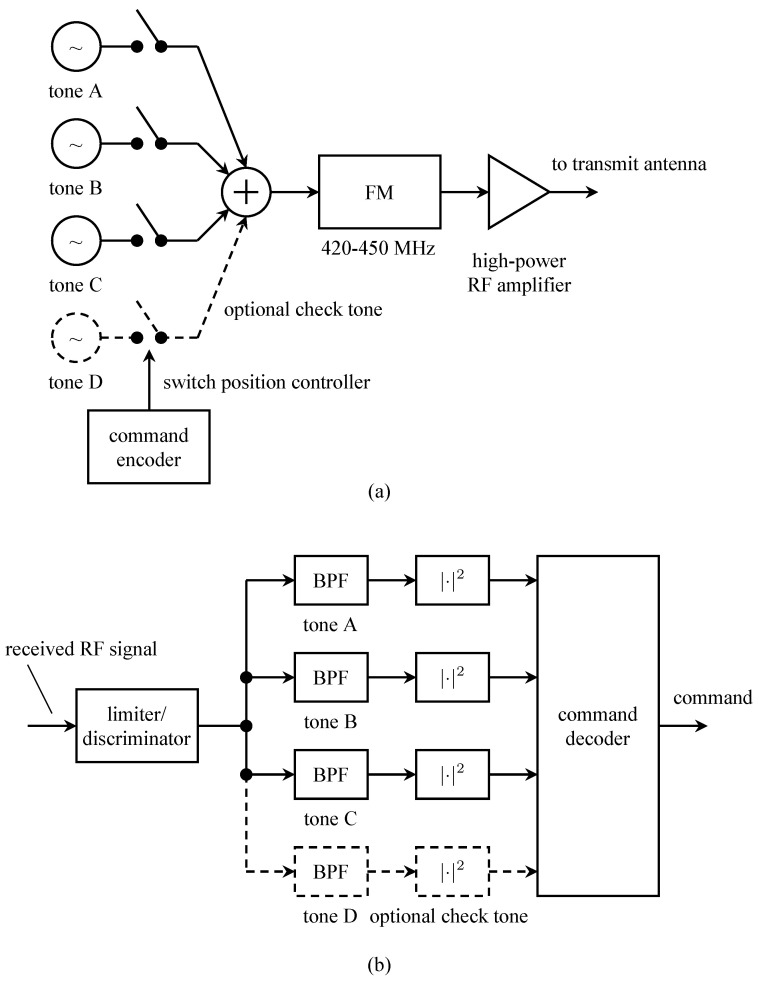
A block diagram of the FTS dating from the 1950s: (**a**) the (ground-based) transmitter; (**b**) the (airborne) demodulator/decoder.

FTS dates from the early 1950s [190] and is based on a series of combinations of three or four tones modulating an FM carrier in the 420 to 450 MHz band. A block diagram of the transmitter is shown in Figure 23a. The three tones, used to define four commands, are drawn from a set of 20 RCC tones in the range 7.5 kHz to 73.95 kHz. The optional fourth tone provides a check channel. The tones are either on or off, depending on the command. The sum of the tones is used to modulate the FM carrier. A block diagram of the flight termination receiver is shown in Figure 23b. The received signal is applied to a limiter/discriminator to produce a noisy version of the three/four-tone combination. The power at each of the tone frequencies is monitored by the command decoder that identifies the uplink command.

While primitive by today’s standards, the FTS worked well for almost 50 years. FTS was an example of the well-worn adage, “if it ain’t broke, don’t fix it”. In March 1999, test flights with the then-new Global Hawk [191] unmanned air vehicle broke FTS [192,193]. The Global Hawk event occurred on the last day of its tests at Edwards AFB, CA, USA. Because the Global Hawk was scheduled for tests at Nellis AFB (just north of Las Vegas, NV, USA) the next day, the Nellis AFB range safety office, following standard procedure, tested the Nellis AFB flight termination system by transmitting a flight termination sequence and monitoring the transmitted signal. The Global Hawk flying high above the Edwards AFB range received the command, successfully decoded it, and terminated by crashing into the desert on the Edwards AFB range. The post-event analysis identified two issues: (1) the Edwards AFB FTS and the Nellis AFB FTS operated on the same carrier frequency and (2) there is no provision in FTS for source and destination addresses. The first issue was caused by the fact that in normal circumstances Edwards AFB and Nellis AFB are too far away from each other for simultaneous line-of-sight RF propagation to/from a test article on the Edwards AFB range. Global Hawk’s high-altitude operation violated this assumption. For the second issue, if FTS had a provision for even simple source and destination addressing, the accident would not have occurred even without the resolution of the first issue.

The second observation prompted a program called the *enhanced* flight termination system (EFTS) in April 2000 [190,194,195]. The result was a system that provided source/destination addressing, enhanced reliability through channel coding, and increased security through encryption. The last two items nudged the design toward a “digital system” and away from a modified tone-based system, such as the one described in [196].

**Figure 24:**
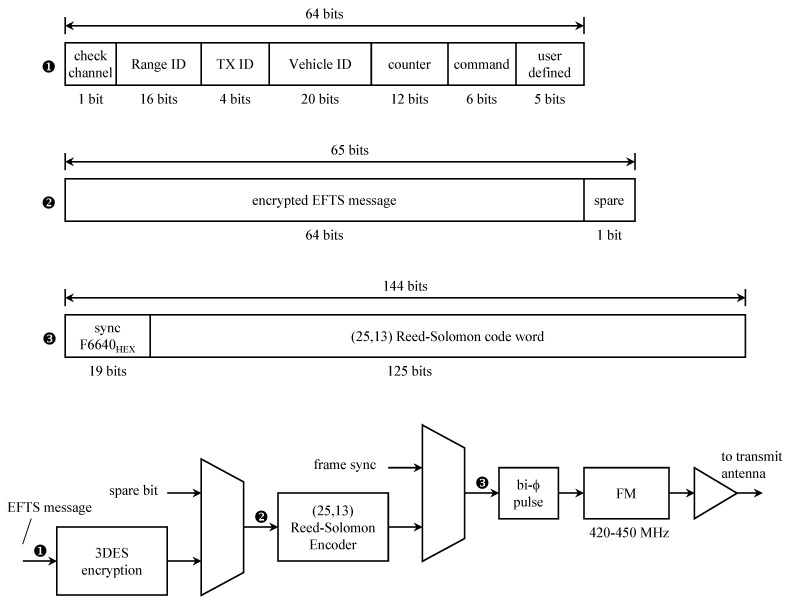
A block diagram of the EFTS transmitter.

The result was the system summarized by the transmitter block diagram in Figure 24 [197]. The EFTS message includes test range, transmit, and vehicle identification codes with the 6-bit command code as shown. After encryption (and the insertion of a spare bit to match the code dimension), the encrypted message was encoded by a (25,13) Reed–Solomon code with elements from GF $\left(2^{5}\right)$. The code was obtained from the (31,13) Reed–Solomon code by puncturing. After the addition of a 19-bit frame synchronization pattern to identify the start of the codeword, the message is transmitted using bi-ϕ PCM/FM. Messages are sent continuously at a rate of 50 messages/s, equivalent to an over-the-air bit rate of 7.2 kbits/s. Because a secondary goal was to reuse as much of the installed FTS infrastructure as possible, the bi-ϕ pulse was used to remove the DC component from the FM modulator input because high-power FM modulators are usually AC-coupled to their inputs. The frequency deviation was set to a rather large value of 300 kHz (the instantaneous frequency was the carrier ± 300 kHz) to compensate for receiver/demodulator phase noise caused by shock and vibration on the airborne test article.

EFTS was successfully tested at Eglin AFB, FL, USA, and Tyndall AFB, FL, USA in 2007 [198]. In the end, EFTS was not included in the IRIG 319 Standard. Just after the EFTS tests, the number of experimental UAVs that required testing declined. Accompanying this decline was the introduction of autonomous flight termination systems (AFTS) [199,200] primarily for weapons and launch vehicles. Together, these trends reduced the perceived need for the advantages of EFTS over FTS [201].

#### 4.2.2. IRIG 106 LDPC Codes

Low-density parity check (LDPC) codes were introduced in the IRIG 106 telemetry standard in 2015 [202]. The IRIG 106 LDPC codes are the family of nine AR4JA LDPC codes adopted in the CCSDS standard [50] and described above in Section 3.10. The codes were designed for use with BPSK. Consequently, the LDPC codes are only defined for use with SOQPSK-TG in IRIG 106. The reason the AR4JA code works with SOQPSK-TG is because SOQPSK-TG is a CPM with modulation index h=1/2; CPMs with h=1/2 often “behave like QPSK” which, in turn, “behaves like BPSK”. The code parameters are listed in Table 2.

Code performance, measured by the decoded bit error rate displays approximately 0.5 dB difference between k=1024 and k=4096. The rate-2/3 code is about 0.75 dB worse than the rate-1/2 code, and the rate-4/5 code is about 1 dB worse than the rate-2/3 code. See Figure D-10 of [163].

**Figure 25:**
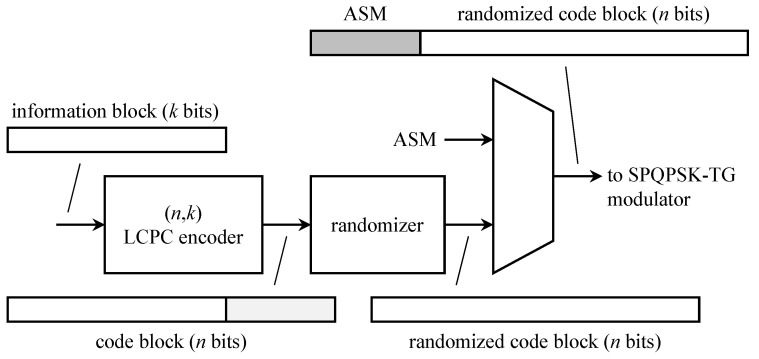
A block diagram of the encoding procedure for the IRIG 106 LDPC codes.

The encoding procedure is illustrated in Figure 25. Because the data and the resulting codeword do not guarantee sufficient transitions to achieve symbol timing synchronization, the codeword is randomized. To aid in synchronization, an attached sync marker (ASM) is prepended to each codeword. The ASM is a 64-bit sequence for k=1024 and a 256-bit sequence for k=4096. As of this writing, the IRIG 106 LDPC codes have been used in a number of tests. See [203,204,205,206,207,208].

#### 4.2.3. iNET

Integrated Network Enhanced Telemetry (iNET) was introduced in the 2017 version of IRIG 106 [209] and defined a wireless networking approach to aeronautical mobile telemetry. iNET was a response to decreasing spectrum and increasing demand for bandwidth. The fundamental idea is that not all test measurements need to be downlinked for every test point. A test point is often defined by a maneuver that stresses certain structural elements. Thus, only the measurements from the stressed structural elements (along with a set of other measurements critical to the safety of flight) need to be downlinked during a test point. Downlinking only the necessary measurements reduces the telemetry bit rate which in turn reduces the RF bandwidth.

**Figure 26:**
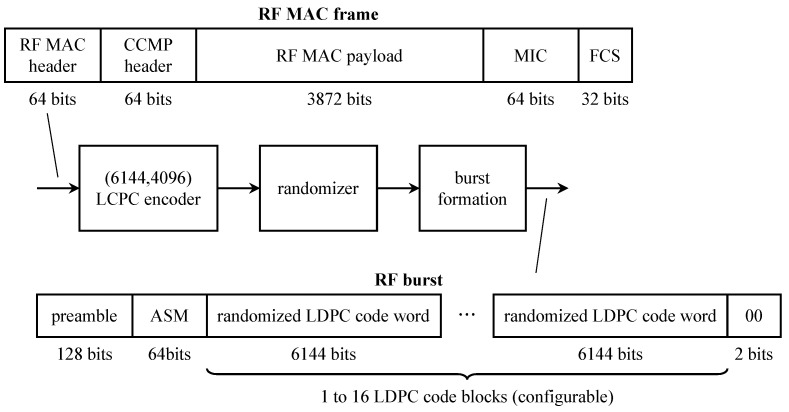
The structure of the iNET RF MAC frame and the RF burst. Adapted from Chapter 27 of IRIG 106-23 [163].

The enabling technologies for iNET were advanced onboard recording equipment, a two-way RF radio link between the ground station and the test article, and a wireless networking protocol. The wireless networking protocol is defined by the Telemetry Network Standard (TmNS) in Chapter 27 of IRIG 106 [163]. TmNS uses time division multiple access (TDMA) based on RF bursts. The format of an RF burst is illustrated in Figure 26. The RF burst comprises a preamble, attached sync marker (ASM), up to 16 randomized LDPC code words, and two tail bits (zeros). The length-128 preamble is a 16-bit pattern repeated 8 times and the ASM is the 64-bit ASM described in Section 4.2.2. The (6144,4096) LDPC code discussed in Section 3.10 (see Table 2) is used to encode an RF MAC frame. The two tail bits are used to place the SOQPSK-TG modulator in the all-zeros state at the end of the burst. This is done to improve the performance of trellis-based detection [184].

The input to the LDPC encoder is an RF MAC frame whose structure is also shown in Figure 26. The last 32 bits are the frame check sequence (FCS) field and are computed from the length-32 CRC code defined by the polynomial (15). The check bits are used to protect the MAC frame from LDPC decoder errors.

The modulation is SOQPSK-TG. The coded (over the air) bit rate is 20 Mbits/s during a burst. Because the LDPC code is a rate-2/3 code, the information bit rate is 13 13 Mbits/s. As of this writing, iNET is being actively developed and tested for future use.

#### 4.2.4. Packet Telemetry Downlink

Packet telemetry downlink is a provision in IRIG 106 that facilitates the inclusion of packet data, such as Ethernet packets, in continuous framed PCM transmissions, TmNS messages (see Section 4.2.3), or “Chapter 11” packets (see Chapter 11 of IRIG 106-23 [163]). When used, the data occupies one minor frame of the PCM stream. (This is a generalization of the simple PCM example illustrated in Figure 21). A preliminary version appeared in Chapter 7 of the 2015 version of IRIG 106-15. The current fully-developed version first appeared in Chapter 7 of the 2017 version of IRIG 106-17 [209].

**Figure 27:**
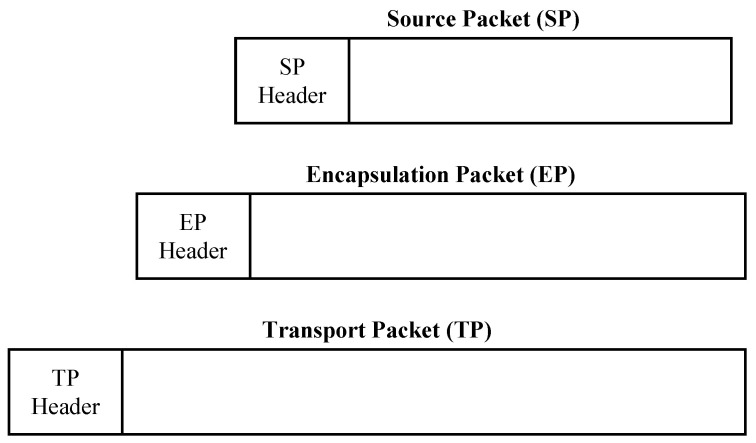
The three packet layers defined in the packet telemetry downlink standard in IRIG 106, Section 7 [163].

The standard defines three packet layers illustrated in Figure 27. The packet produced by its source is called a source packet (SP). These packets usually come with their own headers. Each SP is encapsulated in an encapsulation packet (EP). Finally, the EP is encapsulated in a fixed-length transport packet (TP). The illustration in Figure 27 is simplified for illustration. SPs are usually not the same length as a PCM minor frame. For example, when an SP is longer than a minor frame, it is partitioned into an integer number of EPs.

The packet type and its length are identified in its header (the SP Header in Figure 27). The EP header also encodes the packet type and its length. The TP header contains sequencing information. Because errors in the SP, TP, and EP headers can lead to lengthy data outages, critical information in these headers are protected by the (24,12) Golay code. The Golay code, in systematic form, is defined by the generator matrix

(16) $$G=\left[\begin{array}{c} 1\;0\;0\;0\;0\;0\;0\;0\;0\;0\;0\;0\;\;1\;1\;0\;0\;0\;1\;1\;1\;0\;1\;0\;1\\ 0\;1\;0\;0\;0\;0\;0\;0\;0\;0\;0\;0\;\;0\;1\;1\;0\;0\;0\;1\;1\;1\;0\;1\;1\\ 0\;0\;1\;0\;0\;0\;0\;0\;0\;0\;0\;0\;\;1\;1\;1\;1\;0\;1\;1\;0\;1\;0\;0\;0\\ 0\;0\;0\;1\;0\;0\;0\;0\;0\;0\;0\;0\;\;0\;1\;1\;1\;1\;0\;1\;1\;0\;1\;0\;0\\ 0\;0\;0\;0\;1\;0\;0\;0\;0\;0\;0\;0\;\;0\;0\;1\;1\;1\;1\;0\;1\;1\;0\;1\;0\\ 0\;0\;0\;0\;0\;1\;0\;0\;0\;0\;0\;0\;\;1\;1\;0\;1\;1\;0\;0\;1\;1\;0\;0\;1\\ 0\;0\;0\;0\;0\;0\;1\;0\;0\;0\;0\;0\;\;0\;1\;1\;0\;1\;1\;0\;0\;1\;1\;0\;1\\ 0\;0\;0\;0\;0\;0\;0\;1\;0\;0\;0\;0\;\;0\;0\;1\;1\;0\;1\;1\;0\;0\;1\;1\;1\\ 0\;0\;0\;0\;0\;0\;0\;0\;1\;0\;0\;0\;\;1\;1\;0\;1\;1\;1\;0\;0\;0\;1\;1\;0\\ 0\;0\;0\;0\;0\;0\;0\;0\;0\;1\;0\;0\;\;1\;0\;1\;0\;1\;0\;0\;1\;0\;1\;1\;1\\ 0\;0\;0\;0\;0\;0\;0\;0\;0\;0\;1\;0\;\;1\;0\;0\;1\;0\;0\;1\;1\;1\;1\;1\;0\\ 0\;0\;0\;0\;0\;0\;0\;0\;0\;0\;0\;1\;\;1\;0\;0\;0\;1\;1\;1\;0\;1\;0\;1\;1 \end{array}\right]$$

The standard modifies the SP headers for TmNS messages and Chapter 11 packets. The modified Chapter 11 SP Header comprises 4 (24,12) Golay code codewords followed by 96 bits (12 bytes) of unprotected information. The modified TmNS message SP Header comprises 8 (24,12) Golay code codewords plus a variable number of unprotected bits. The standard does not modify the SP header for Ethernet packets. The EP Header comprises 2 (24,12) Golay code codewords and no unprotected bits. The TP comprises one (24,12) Golay code codeword plus 8 bits (1 byte) of unprotected data. The (24,12) Golay code was chosen because encoding and decoding algorithms were readily available, simple to implement, and the right size for protecting critical header information [210,211].

Additional coding to protect the payload portion of the PT data frames was considered when the standard was being written. The length-255 Reed–Solomon codes defined in CCSDS 131.0.B-2 [50] was of special interest [210,211]. Additional coding was not adopted in the standard due to the encryption issue described in Section 2. When the IRIG 106 LDPC codes described in Section 4.2.2 became commonly available, the need for additional coding in SOQPSK-TG systems diminished [211]. However, for PCM/FM systems (where LDPC codes are currently not available), at least one manufacturer applies length-255 CCSDS Reed–Solomon codes to the PTFR payload data [211,212].

## 5. Summary and Conclusions

The journey through the history of channel coding in deep-space telemetry began with codes for which practical soft-decision decoding algorithms were known: the (32,6) Reed–Muller code for *Mariner* 6 and the (25,12) convolutional code for *Pioneer* 9. The decoder for the *Pioneer* 9 convolutional code was a sequential decoder based on the Fano algorithm. Subsequent *Pioneer* missions used longer convolutional codes designed for decoding using the Fano algorithm to increase coding gain. The invention of the Viterbi algorithm made possible soft-decision maximum likelihood sequence detection of convolutional codes with modest constraint lengths. The Viterbi algorithm with the shorter constraint length convolutional codes achieved higher coding gains than the *Mariner* convolutional codes with sequential decoding. The (7,12) convolutional code was used for the *Voyager* and *Viking* programs. The *Voyager* program introduced concatenated coding as a way to further improve coding gain without the exponential increase in decoder complexity. Both the (7,12) convolutional code and its concatenation with the (255,233) Reed–Solomon code became international standards for deep-space telemetry. Creative use of channel coding coupled with an early form of iterative decoding allowed the *Galileo* mission to achieve 70% of its mission goals despite a series of near-devastating catastrophes. The introduction of turbo codes in the 1990s spawned a new era in channel coding for deep-space telemetry. The turbo code concept was capable of producing low-rate codes with manageable decoding complexity. These codes were essential to deep-space missions to the Kuiper belt and higher data rate missions closer to home.

In aeronautical mobile telemetry, the adoption of channel codes took longer. In the beginning, telemetry links used analog modulations on the RF links; digital modulation, a prerequisite for the application of channel coding, was not fully adopted until 15 years after space telemetry had adopted it. Even after the transition to fully digital transmission formats, channel coding was not essential for closing the link in most aeronautical mobile telemetry applications. For test points that produced low-quality data, the test point is usually re-flown, a sort of high-level automatic repeat request (ARQ) system with humans in the loop. Channel coding held the promise of reducing the number of test points that had to be re-flown, but in spectrally congested areas, the bandwidth expansion that accompanies the use of channel coding was seen as an unacceptable cost. There were edge cases where channel coding was needed, and these edge cases kept channel coding “in the discussion” until good codes compelled their adoption.

By the time channel codes received serious consideration, the reduction in available spectrum coupled with an increase in required data rates pushed spectral efficiency to the top priority. Non-binary modulations were adopted and high-rate codes were sought. Because high-rate block codes tend to perform better than high-rate convolutional codes, a family of high-rate LDPC codes was adopted by the aeronautical mobile telemetry community (this was the same family of LDPC codes adopted by the deep-space telemetry community a few years earlier).

The channel coding history illustrates the ingenuity and creativity of dedicated engineers and scientists. The early motivation for coding was based solely on mathematical analysis, not on experience. The fact that channel coding became such an enabler for deep-space telemetry is a testament to the correctness of the mathematical models and the collaborations required to achieve success in extraordinarily complex undertakings. In many ways, deep space was the perfect setting for channel coding to evolve from a mathematical curiosity to an essential component with supporting hardware. To all who worked so hard to apply channel coding to telemetry, the author gives a hearty “well done”!

## Figures and Tables

**Table 1:** The relationship between the turbo code rate and the constituent code output connections. Adapted from [50].

Turbo Code Rate	Turbo Code Outputs
1/2	out 0a, out 1a, out 0a, out 1b, …, repeated (k+4)/2 times
1/3	out 0a, out 1a, out 1b, …, repeated k+4 times
1/4	out 0a, out 2a, out 3a, out 1b, …, repeated k+4 times
1/6	out 0a, out 1a, out 2a, out 3a, out 1b, out 3b, …, repeated k+4 times

**Table 2:** CCSDS LDPC code parameters.

	Rate-1/2	Rate-2/3	Rate-4/5
k=1024	n=2048	n=1536	n=1280
k=4096	n=8192	n=6144	n=5120

## Data Availability

No new data were created or analyzed in this study. Data sharing is not applicable to this article.
